# Thiol-ene click reaction as an effective tool for the synthesis of PEG-functionalized alkoxysilanes-precursors of anti-fog coatings

**DOI:** 10.1038/s41598-023-48192-4

**Published:** 2023-11-29

**Authors:** Marta Kaczmarek, Agnieszka Przybylska, Anna Szymańska, Agnieszka Dutkiewicz, Hieronim Maciejewski

**Affiliations:** 1 Faculty of Chemistry, Adam Mickiewicz University, Uniwersytetu Poznańskiego 8, 61-614 Poznań, Poland; 2 Poznań Science and Technology Park, Adam Mickiewicz University Foundation, Rubież 46, 61-612 Poznań, Poland

**Keywords:** Chemical synthesis, Materials science

## Abstract

The article presents a very simple method of glass modification to obtain the anti-fog effect. Silanes containing two types of functional groups, namely a hydrophilic and polar polyether group and an alkoxysilyl group (to bond with the surface of the modified material) were synthesized in thiol-ene reactions. The hydrothiolation reactions of polyethers containing a C=C terminal bond with mercaptoalkoxysilane proceeded efficiently, yielding quantitatively appropriate products under mild reaction conditions. This method enabled the synthesis of a series of alkoxysilanes functionalized with polyethers, differing in structure. The group of obtained derivatives was characterized by ^1^H, ^13^C, ^29^Si NMR, and FT-IR analyses, and then used to prepare coatings on glass using the sol–gel method. The coated glass surfaces exhibited transparency, superhydrophilic or hydrophilic properties, anti-fog and anti-frost performance.

## Introduction

Fogging of transparent surfaces made of glass, PC, or PET causes a number of negative effects related to the loss of light transmission, image blurring, and limited or no visibility. This phenomenon is not only annoying but can in some cases be very dangerous^1,2^. In particular, it is a big problem in the automotive and aerospace industries (windows and rear-view mirrors), refrigeration (food packaging), photovoltaics (solar cells), medical analytics (laparoscopes and microscopes), agriculture (greenhouses), and in many aspects of everyday life (glass and bathroom mirrors, goggles, glasses, camera lenses, etc.)^1,3–5^.

One of the most common and effective methods of obtaining an anti-fog (anti-dew) surface is to cover the surface of the transparent material with a special coating. Such coatings, due to their wettability, are divided into (super)hydrophobic and (super)hydrophilic^6,7^. (Super)hydrophobic coatings reduce adhesion and increase the repulsion of water droplets from the substrate, resulting in water droplets rolling off the surface. However, (super)hydrophobic coatings are often not transparent and require a surface with a suitable morphology. On the (super)hydrophilic surface, the condensing vapor dissolves quickly, forming a homogeneous, pseudo-film that prevents the formation of fog and frost. Superhydrophilic coatings are still being intensively researched^8–11^. A variety of compounds containing polar functional groups with high surface energy, such as hydroxyl (OH), amino (NH_2_), amide (NHCOR), carboxylic/ester (COOH/COOR), sulfonic (–SO_3_H), and dihydrogen phosphate (PO_4_H_2_) are often used to create a superhydrophilic surface^12,13^. (Super)hydrophilic coatings are highly desirable in many other important applications, i.e., antireflective, antifouling, self-cleaning, or bioactive materials and so on^14,15^. Briscoe and Galvin ^16^, based on theoretical calculations, showed that there are no transmittance losses for surfaces with a water contact angle below 40°. As the WCA increases to 90°, the transmittance decreases from about 90% to 50%. In this way, the surface haze of the material can be reduced by modifying it to obtain a WCA of less than 40–50°. However, there are natural and synthetic polymers that, thanks to their absorbent or self-cleaning properties, can be used to obtain optically transparent coatings in fog conditions, for which WCA ≥ 50°^17^.

Various strategies are used to produce hydrophilic coatings. One of the most effective and widely used methods of modifying glass surfaces is the sol–gel process^18,19^. This method has many advantages, including simplicity of the process, the ability to synthesize inorganic–organic hybrid materials, mild processing conditions, ease of application to the surface, and the ability to synthesize amorphous materials in thin layers. In addition, by adjusting the synthesis parameters, it is possible to control the functionality, morphology, and microstructure of the surface^19–22^.

Many organofunctional silanes, containing different functional groups can be used to create hybrid materials. The earlier work of our team enabled the development of a polysiloxane with two types of functional groups, a polyether as a hydrophilic part (giving anti-fog properties) and alkoxysilyl groups, enabling bonding with substrates^23^. Polyether moieties are characterized by excellent hydrophilic properties, transparency, and flexibility^24,25^. The oxygen atoms in the polyether chain interact with the glass surface through hydrogen bonds, thus stabilizing the coating^23^. There are known coatings containing perfluoroalkyl groups and poly(ethylene glycol) (PEG) segments exhibiting hydrophilic and oleophobic properties, making them self-cleaning and anti-fog coatings^26^. Maeda et al. developed an anti-fog coating based on polysilsesquioxane containing tetraethylene glycol chains, obtained in a sol–gel reaction. Tetraethylene glycol chains have been found to improve coating durability^27^. The introduction of oligo(ethylene glycol) (OEG) moieties into the coating may add frost-resistant capability. OEG can strongly interact with the water molecules and inhibit frost formation during condensation, which was confirmed by Yoon et al^28^.

The main goal of this work was to design and synthesize compounds that would allow simple and inexpensive modification of the glass surface to obtain the anti-fog effect. An additional objective was to determine the effect of the structure of the compounds used on the hydrophilicity and anti-fog properties of the coated glass surfaces. Alkoxysilanes containing a polyether chain were used to modify the glass surfaces, which were deposited using the sol–gel method. These compounds were obtained by thiol-ene click reaction. To the best of our knowledge, the use of polyether group-containing alkoxysilanes obtained by the thiol-en-click reaction as anti-fog glass surface modifiers has not yet been described.

## Methods

### Materials

Allyl polyethers and esters were purchased from ICSO Chemical Production (Poland). 2,2-Dimethoxy-2-phenylacetophenone (DMPA) and CDCl_3_ were purchased from Aldrich. 3-Mercaptopropyltrimethoxysilane (MPTMS) was purchased from ABCR. Methanol, acetic acid, HCl, and NH_4_OH were purchased from POCh. Glass plates (25.4 mm × 76.2 mm and 1.0–1.2 mm thickness) were purchased from Bionovo.

### Preparation of PEG-functionalized trimethoxysilanes

**Figure 1:**
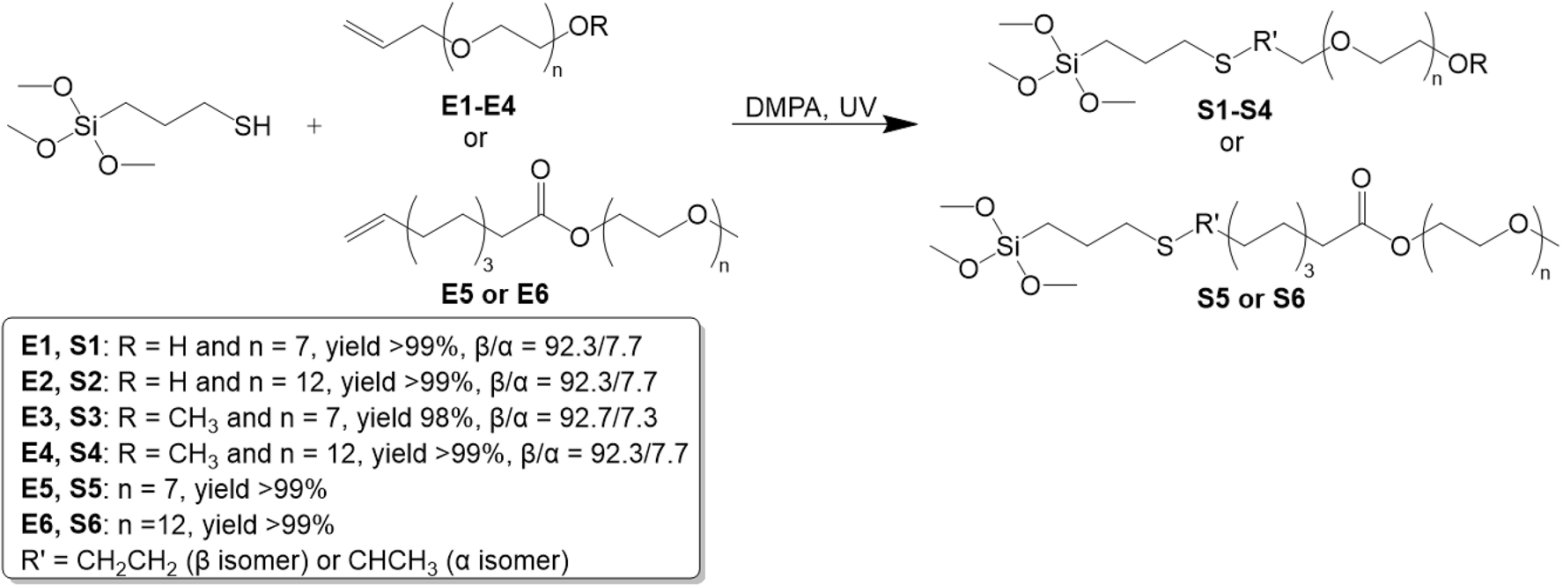
Synthesis of PEG-functionalized trimethoxysilanes via hydrothiolation reaction.

The PEG trimethoxysilanes (S1–S6) were synthesized via hydrothiolation reaction between 3-mercaptopropyltrimethoxysilane and unsaturated carbon-carbon double bond of allyl polyethers (E1-E4) or esters containing polyether and terminal HC=CH_2_ units (E5 and E6) in the presence of photoinitiator DMPA under UV irradiation, according to Fig. 1.

Typically MPTMS, E1-E6, and DMPA, at the molar ratio of 1.00:1.00:0.01, respectively, were placed in a glass vessel and the mixture was irradiated for 1 h without solvent, at room temperature. Products were analyzed without further purification by ^1^H, ^13^C, ^29^Si NMR, and FT-IR spectroscopy (characterization of compounds and NMR spectra are available in Supplementary Information).

### Preparation of modifying solutions

The solutions were prepared based on the previously reported procedure^29^. A mixture consisting of 0.007 mol of compound (S1–S6), 9.74 ml of methanol, and 0.5 ml of acetic acid solution (0.001 M) was stirred for 30 min and left for 24 h at room temperature. After this time, 0.61 ml of ammonium hydroxide solution (11.2 M) was added to the mixtures, and following 15 min of stirring, the solutions were left for 2 days for condensation at room temperature. The resulting formulations were diluted with 10 ml of methanol and stirred for 15 min. The coatings were applied to the glass plates immediately after 2 days of condensation, as well as after 3, 5, 10, 20, and 31 days of aging (storing the solution in a laboratory environment (~22 °C and 35–45% relative humidity (RH))).

### Deposition of the coatings onto glass plates

The glass slides were cleaned in a solution of methanol and HCl (1:1, v/v) for 30 min, then rinsed with distilled water and left to air dry. A piece of paper towel was soaked with 0.5 ml of the prepared solution and the coating was applied by rubbing each side of the glass plate twenty times. The prepared coatings were left to dry overnight at room temperature.

### Analytical methods

Nuclear magnetic resonance spectra ^1^H, ^13^C, and ^29^Si NMR were recorded at 298 K on Bruker Avance III HD 400 MHz spectrometers. CDCl_3_ was used as a solvent. Fourier transform infrared (FT-IR) spectra of the polymers were taken on a Bruker spectrometer, model Tensor 27, equipped with a SPECAC Golden Gate diamond ATR (Attenuated total reflection) unit. In each case, 16 scans were collected for a spectrum at the resolution of 2 cm^-1^. Static water contact angle measurements were made using an automatic video contact-angle testing apparatus, Krüss GmbH, model DSA 100 Expert, equipped with a software-controlled (DAS4 2.0): measuring table (position in x, y, and z-axis), automatic, four-channel dosing unit and 780 × 580 pixel camera with adjustable focus, contrast, exposure time and illuminance to confirm the hydrophilic nature of the modified substrates. A 5 μL volume of water droplet profile was isolated in real-time based on a circle fit model from images obtained at a 0° camera tilt. The presented water contact angle values are the arithmetic mean of measurements for 5 drops. The contact angles for each drop were also determined as the average of 10 measurements (5 measurements per second for 2 s) made 3 s after their deposition on the surface of the tested sample. The visible light transmittance of the samples was collected using a UV-vis spectrophotometer (Jenway 6715), with the wavelength range from 400 nm to 700 nm.

### Antifogging properties of obtained coatings

Each of the modified glass plates and the unmodified reference plate were placed over a beaker, 1.5 cm above the hot water (~65 °C, 100% relative humidity (RH)) for ~15 s and photographed. These tests were performed using glass slides stored at room temperature and after 1 h of storage in the refrigerator (3 °C) and freezer (− 20 °C). All photographs are available in Supplementary Information.

To quantitatively evaluate the anti-fog and anti-frost properties of the coatings the light transmittance in the range of 400–700 nm was recorded on a UV-vis spectrophotometer during the anti-fogging and anti-frosting experiments. In anti-fogging experiments, light transmissions were measured immediately after 15 s exposure to hot water vapor (5 cm above 80 °C water, 100% RH) under ambient lab conditions (temperature 22 °C, 38% RH). In anti-frosting experiments, light transmissions were measured immediately after 30 min storage in the freezer at − 20 °C, after transfer to ambient lab conditions (temperature 22 °C, 38% RH).

To check the durability of the prepared coatings, their WCA values were measured after storing them in laboratory conditions (~22 °C and 35–45% RH) for 1 month (31 days). Additionally, coatings prepared after condensation and stored for a month were exposed to high (60 °C) and low temperatures (− 20 °C) for 3 weeks. WCAs values after 1, 14, and 21 days of storage in the above-mentioned conditions were measured. Anti-fogging properties were tested after 2 months of storage at room temperature.

## Results and discussion

**Figure 2:**
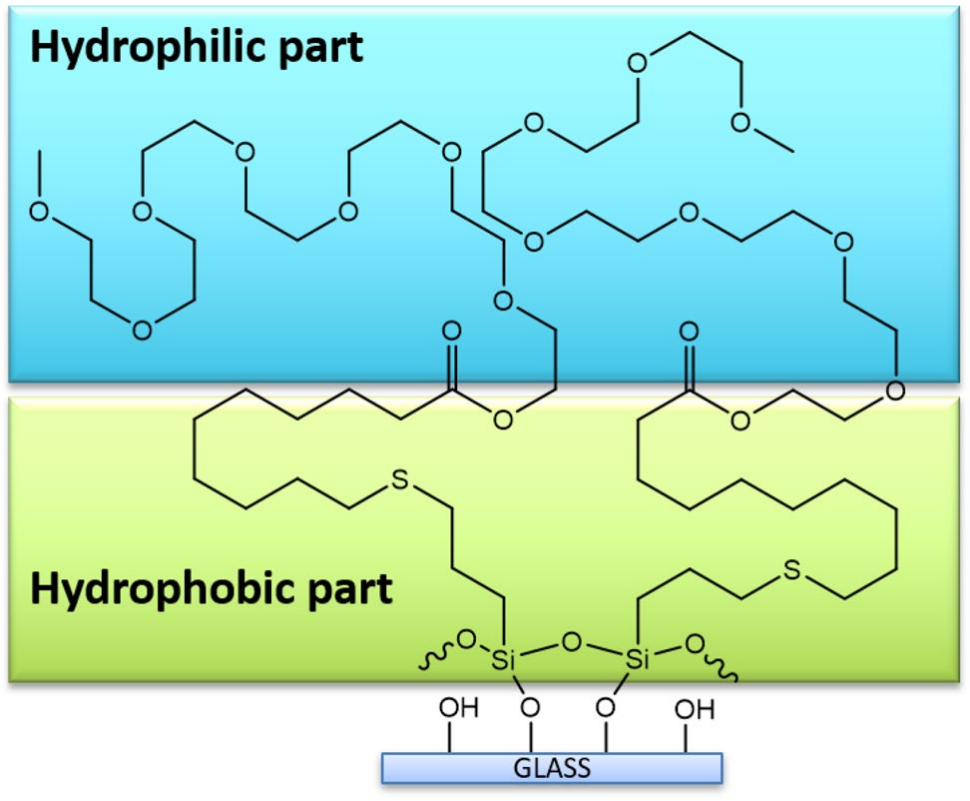
S5-modified glass surface.

During this study, polyether group-containing trimethoxysilanes were used for the preparation of hydrophilic antifogging coatings. Trimethoxysilyl groups present in the discussed derivatives are firstly hydrolyzed by water to silanol groups and next condensed to form a crosslinked network on the glass surface. A schematic illustration of creating of coating on glass is shown in Fig. 2.

A total of 6 organofunctional silanes were synthesized by thiol-ene “click” reaction. A number of compounds were obtained via the addition of 3-mercaptopropyltrimethoxysilane to compounds containing polyethers unit and terminal C=C bond, in a short time (1 h), under mild reaction condition (in bulk, using photoinitiator and UV irradiation). The method of synthesis was simple and efficient, and the products obtained in quantitative yield could be further used without further purification.

The obtained PEG-functionalized trimethoxysilanes (S1–S4) differ in the length of the polyether chains and the terminal group –OH or –OCH_3_. The other two (S5 and S6) contain an additional ester group and a longer alkyl linker in their structure (Table 1).

**Table 1:** Structures of the polyethers used (E1-E6) and the corresponding silanes (S1-S6).

Polyethers		Corresponding silanes	
	E1: n = 7		S1: n = 7
	E2: n = 12		S2: n = 12
	E3: n = 7		S3: n = 7
	E4: n = 12		S4: n = 12
	E5: n = 7		S5: n = 7
	E6: n = 12		S6: n = 12

**Figure 3:**
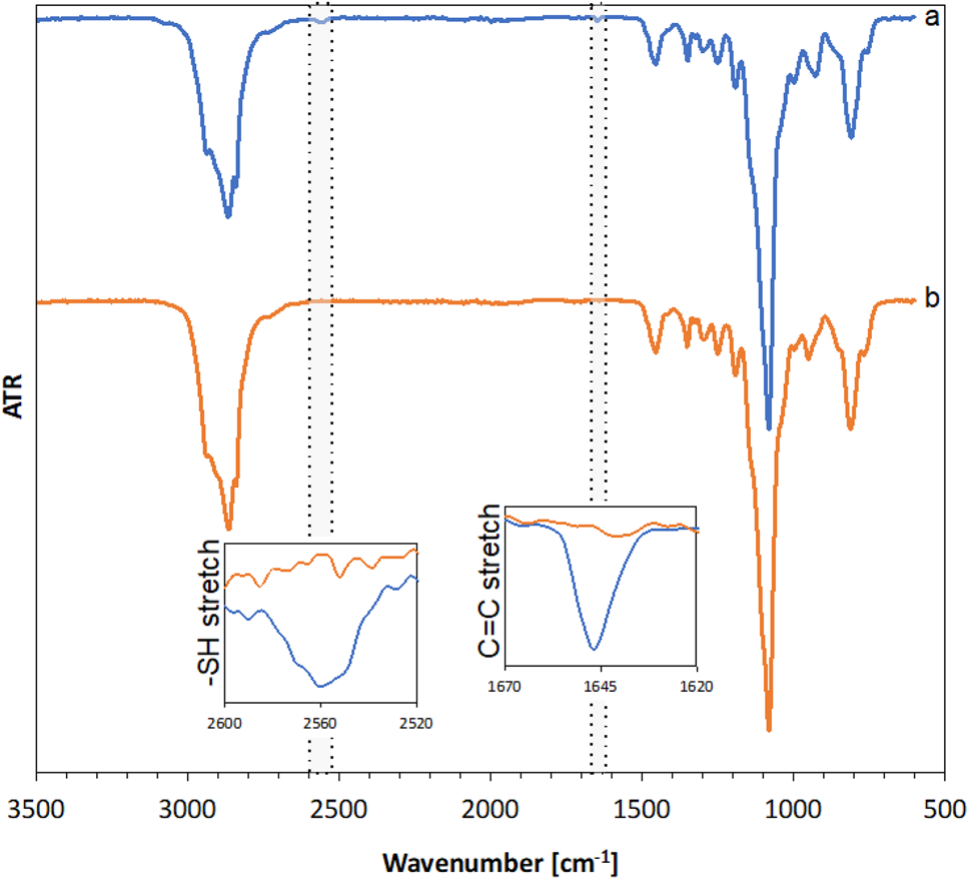
FT-IR spectra of reactants mixture: MPTMS and allyl polyethers (E3) (**a**) and product S3 (**b**).

The synthesis process was monitored by FT-IR spectroscopy, by analyzing a mixture of reactants at time 0 h (before adding the photoinitiator and UV irradiation) and after 1 h. FT-IR exemplary spectra of a mixture of reactants and product after reaction are shown in Fig. 3.

Two analytical peaks are observed, at approx. 2560 cm^−1^ and 1645 cm^−1^. FT-IR spectrum of a mixture of reactants (Fig. 3a) gives a band at 2560 cm^−1^ coming from thiol and a stretching vibration band at 1645 cm^−1^ coming from C=C stretching vibration of polyether. After completing the reaction at 1 h (Fig. 3b) the FT-IR spectrum of the product shows that the characteristic bands disappeared indicating a successful thiol-ene reaction.

**Figure 4:**
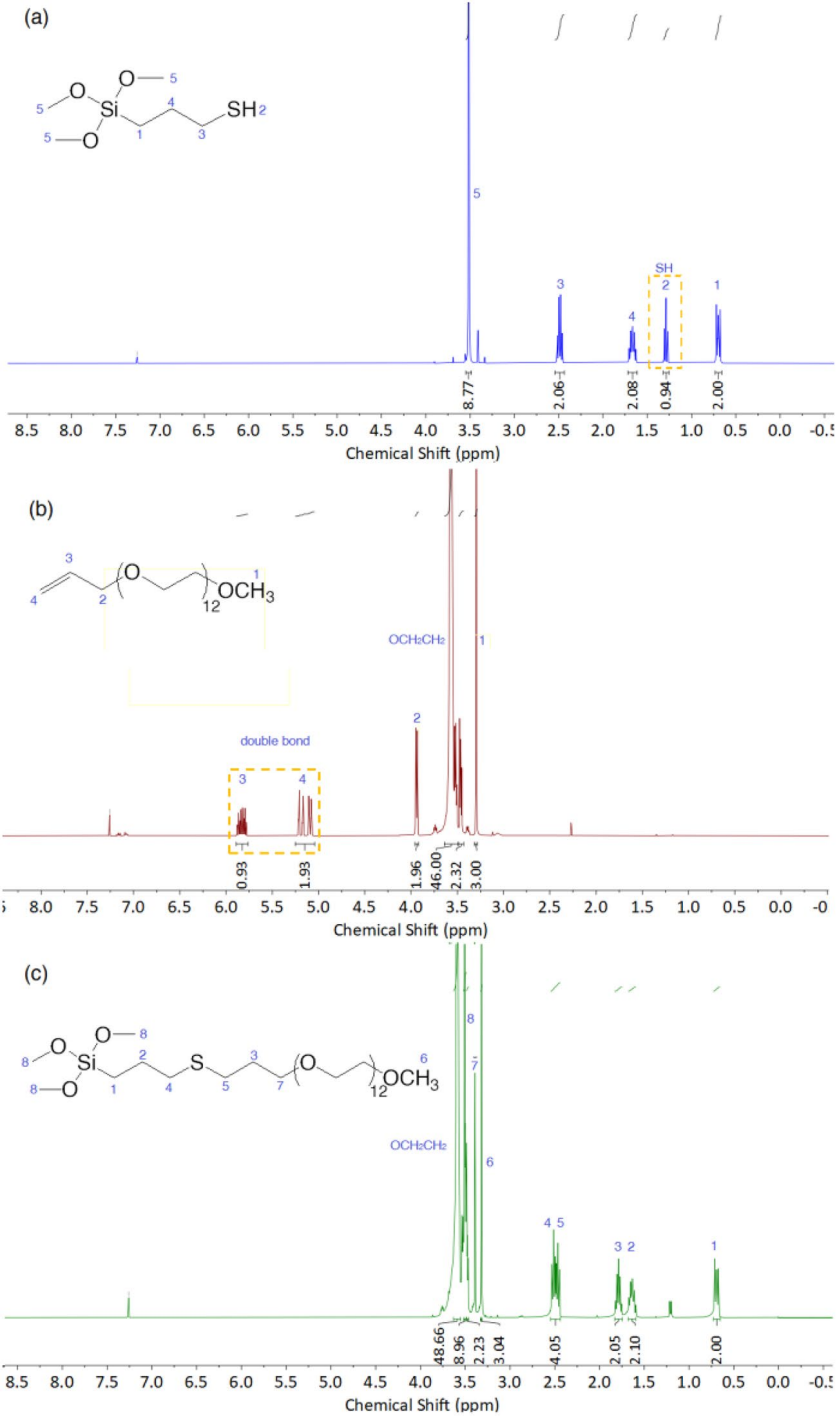
^1^H NMR spectra of (**a**) 3-mercaptopropyltrimethoxysilane (MPTMS), (**b**) E4, and (**c**) product S4.

To verify the thiol-ene reaction had occurred and to confirm the structure of obtained derivatives NMR analyses were recorded. The ^1^H NMR spectrum of a representative sample (derivative S4) is shown in Fig. 4.

The ^1^H NMR spectra of substrates: 3-mercaptopropyltrimethoxysilane (MPTMS) (a) and polyether E4 (b), as well as product S4 (c) with chemical shift assignments are presented in Fig. 4. It can be observed that the triplet at 1.29 ppm (Fig. 4a) attributed to a proton that represents the mercapto group SH and the peak of the double bond in CH_2_=CH (5.83 (m), 5.19 (dq) and 5.09 (dq) ppm, Fig. 4b) vanished entirely in the ^1^H NMR spectra of the product S4 (Fig. 4c). CH_2_SCH_2_ bond formation was confirmed by the appearance of a peak at 2.55–2.45 ppm, which demonstrated that the double bond of E4 had completely reacted with the mercapto group of MPTMS and product S4 was successfully synthesized through the thiol-ene click reaction. The products (S1–S4) contain approximately 7.5% of the α-isomer (for S5 and S6 it was impossible to calculate from ^1^H NMR).

**Figure 5:**
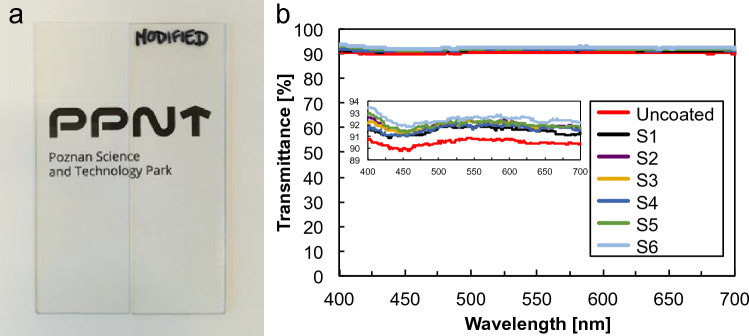
(**a**) Photograph of bare glass plate (left side) and S4-coated glass plate (right side). (**b**) Transmittance curves of the uncoated and coated samples in the wavelength range of 400–700 nm.

Next, formulations were prepared and then deposited on glass plates. Figure 5a shows two glass plates, bare and coated with derivative S4.

There was no visible difference between the modified glass plate (right side) or when compared to the controls (bare glass, left side). The glass substrate with a coating based on S4 exhibited the same excellent optical clarity. Quantitatively, the UV–vis transmission spectra in Fig. 5b revealed that all coated samples are characterized by high visible light transmittance of ~ 92.5%.

**Figure 6:**
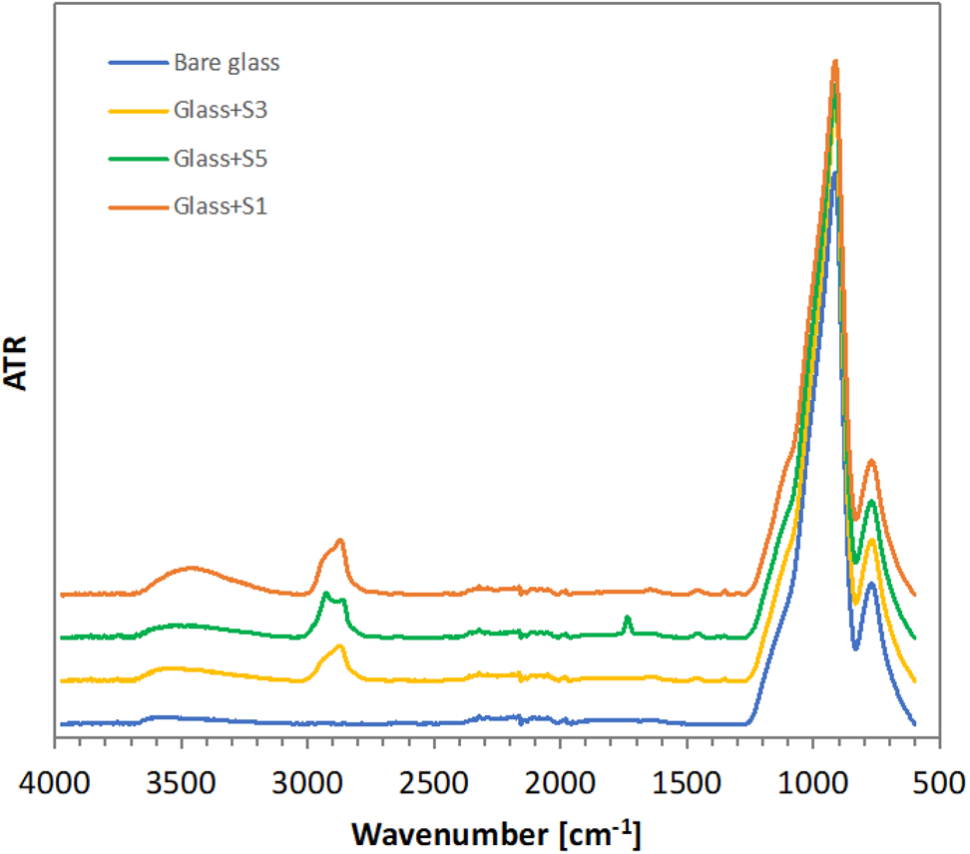
FT-IR of bare glass and glass plates coated with derivatives S1, S3, and S5.

FT-IR spectra of bare glass and glass samples coated with obtained silanes (S1–S6) were recorded. Spectra of bare glass and glass covered with representative derivatives (S1, S3, and S5) are present in Fig. 6.

On all modified samples stretching vibration of the C-H bond appears as a band located at about 2800–3000 cm^−1^ spectral range. Broad IR band between 3700 and 3200 cm^−1^ originating from OH group is more intensive for glass covered with derivative S1 which contains terminal OH group in its structure. On the glass covered with S5, a carbonyl group is observed at about 1745 cm^−1^ and more intensive C–H stretching vibration due to the presence of an additional alkyl linker in its structure. FT-IR spectra of all modified samples confirmed the presence of coatings on prepared glass samples.

The wettability of coatings was investigated by measuring their water contact angles (WCAs). The results are provided in Table 2.

**Table 2:** WCAs of coated samples measured after the modification.

Compound	Uncoated	S1	S2	S3	S4	S5	S6
WCA [°]	28.5 ± 2.0	8.8 ± 0.3	8.5 ± 0.2	8.8 ± 0.6	11.3 ± 0.6	42.0 ± 3.9	38.5 ± 1.6

The water contact angles of glasses covered with derivatives S1-S4 are lower than for uncoated glass, whereas WCAs values of S5 and S6 are higher than the WCA of uncoated glass. All samples are characterized by hydrophilic properties. It was observed that the WCA values were not affected by the length of the polyether chain (S1 vs. S2, S3 vs. S4, and S5 vs. S6) and terminal group –OH versus –OCH_3_ (S1 vs. S3, S2 vs. S4), however, were affected by additional long alkyl group present in compounds S5 and S6. Additionally, hydrocarbon moiety in derivatives S5 and S6 is located between the alkoxysilyl group and polyether chain and has an impact on the character of the coating. Since the hydrocarbon chain exhibits a hydrophobic character, WCA values increase for glass coated with discussed derivatives.

The long-term stability of the covering layer is one of the most important characteristics of coatings. The changes in the WCAs of samples stored in a laboratory environment were the first to be tested. WCAs values of coated glass after preparation and after 1 month of storage in a laboratory environment are shown in Table 3.

**Table 3:** WCAs values of coated samples measured after the modification (1 day) and after storage in a laboratory environment (~ 22 °C and 35–45% RH) for a month.

Compound		S1	S2	S3	S4	S5	S6
WCA [°] after	1 day	8.8 ± 0.3	8.5 ± 0.2	8.8 ± 0.6	11.3 ± 0.6	42.0 ± 3.9	38.5 ± 1.6
	1 month	7.7 ± 1.6	9.0 ± 1.0	7.4 ± 0.1	6.1 ± 1.3	22.8 ± 4.1	16.8 ± 6.3

All coatings are resistant to storage in laboratory conditions. After a month of storage, the glass plates coated with S1-S4 derivatives still retained their superhydrophilic properties, whilst S5 and S6 maintained their hydrophilic character. Interestingly, samples S4, S5, and S6 significantly improved their hydrophilic properties, indicated by the fact that WCA values decreased almost twice after a month (from 11.3° to 6.1° for S4, from 42.0° to 22.8° for S5 and from 38.5° to 16.8° for S6).

The produced coatings consist of a hydrophobic siloxane part and a hydrophilic polyether part. S5 and S6 contain an additional hydrophobic alkyl chain. At the time of preparation of coatings, the long alkyl chains were disordered on the glass surface and the outer alkyl chains resulted in higher WCA value than in the case of S1–S4. Coatings on glass plates act as hygroscopic coatings with high initial WCA. However, as time passed, hydrophilic segments endowed to form the hydration layer on the glass surfaces and WCA values decreased.

**Figure 7:**
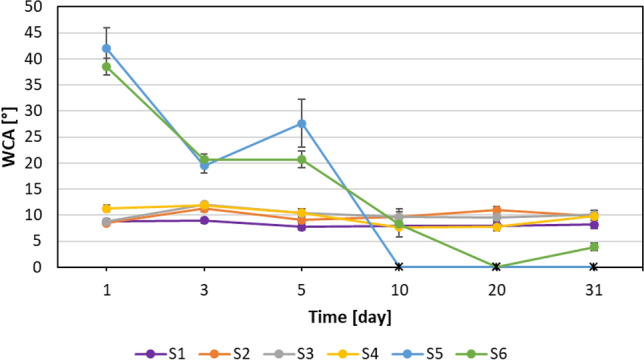
WCAs values of coated samples after storing the solution in a laboratory environment (~ 22 °C and 35–45% RH). * Some WCAs were impossible to measure (the drops of water flowed off immediately). These points are marked on the graph with a black asterisk at 0°.

Another test examined the effect of the aging time of the preparations on the wettability (WCA) of the coated samples. The WCA values of the glass plates coated with formulations on the first day of preparation (day 1 in the graph) and coated with formulations stored for 3, 5, 10, 20, and 31 days were measured. The results are presented in Fig. 7.

Glass samples coated with derivatives S1-S4 still maintain superhydrophilic properties (WCA close to 10°) both after applying the solutions on the day of preparation, as well as after a month of their aging. This is advantageous from the application point of view that solutions can be stored without changing the WCA values. Surprisingly, different results were obtained for coatings prepared from derivatives S5 and S6. Initially, the WCAs values were close to 40°, but as the solutions aged, the WCAs significantly decreased, and coatings prepared after 10 days of aging exhibited a superhydrophilic character and also maintained these properties after a month of aging.

**Figure 8:**
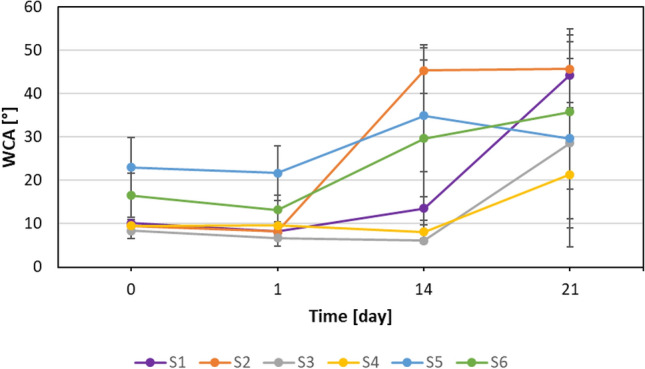
WCAs of coated samples after storage at 60 °C.

Coated glass samples (stored 31 days in ambient lab conditions) were next storage for 3 weeks in very high (60 °C), as well as very low temperatures (− 20 °C). The WCAs of coated samples after storage at 60 °C are shown in Fig. 8.

After 3 weeks of storage glass samples at 60 °C four of them (S3–S6) remain hydrophilic character (WCAs values below 40°). For samples covered with derivatives S1 and S2, a significant increase in WCAs over time (+ 34.2° and + 36.2°, respectively) is observed. This phenomenon can be explained by differences in terminal groups in compounds. Derivatives S1 and S2 contain OH terminal groups as well as S3-S6 contain OCH_3_ terminal groups. WCAs values of glasses covered with S3 and S4 after two weeks are still maintained, even slightly lower than on the date of starting the experiment however, WCAs increase on the 21st day of measurement. In the case of derivatives S5 and S6 WCA values gradually increase during storage, but still retain its hydrophilic properties after 3 weeks. However, it has been observed that the measurement error increases significantly over time. The best result (WCA = 21°) after a 3 weeks of storage at 60 °C was obtained for glass coated with derivative S4 (containing a longer polyether chain and OCH_3_ terminal group).

**Figure 9:**
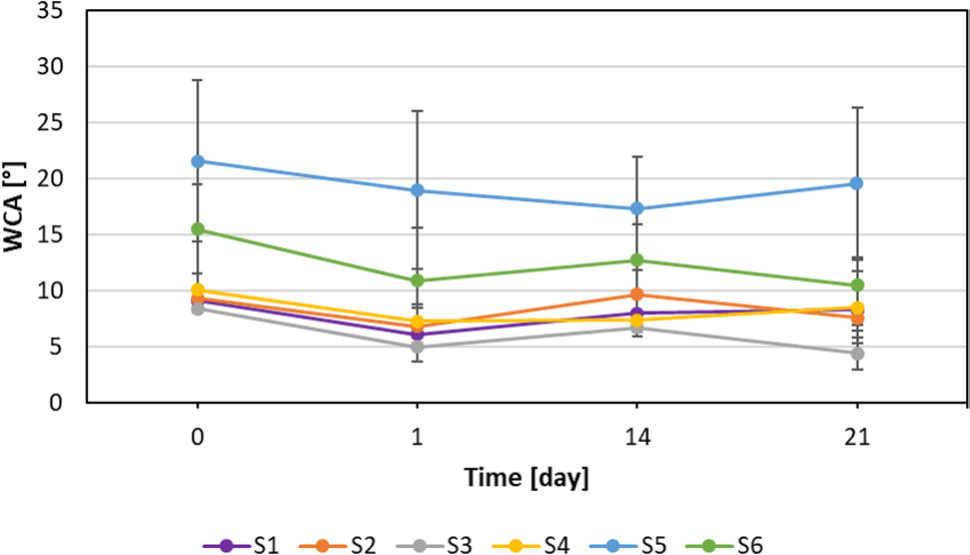
WCAs of coated samples after storage at -20 °C.

WCAs of coated samples after storage at − 20 °C are shown in Fig. 9.

All modified samples are stable in storage at low temperature (− 20 °C). In each case, a slight decrease in values of WCAs was observed after 3 weeks of storage. Four of the samples (S1–S4) retained their superhydrophilic properties, whilst S5 and S6 remained hydrophilic.

**Figure 10:**
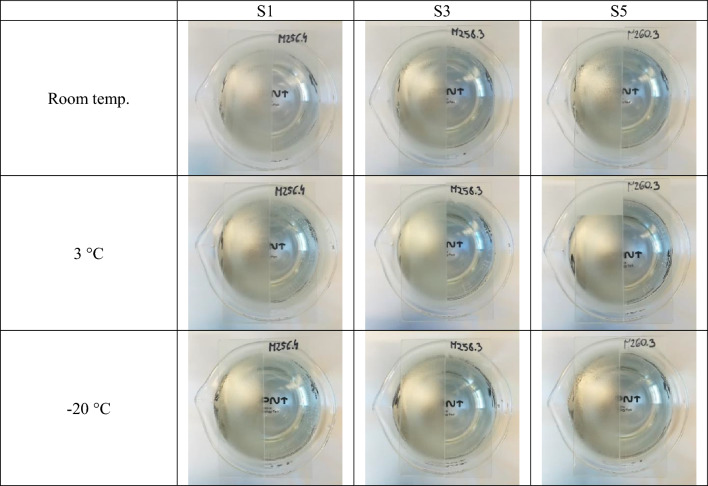
Photographs of uncoated (left side of glass plates) and coated with derivatives S1, S3, and S5 (right side) glass slides after the antifogging test of glasses stored at room temperature and after 1-h storage in the refrigerator (3 °C) and freezer (-20 °C).

Anti-fog tests were carried out by placing two glass plates (raw and modified) on a beaker, 1.5 cm above hot water (~ 65 °C), and observing the settling of water vapor on the plates. These tests were carried out after preparation the formulas as well as after 3, 5, 10, 20, and 31 days of aging of formulations. Photos of the plates after the immediate application of the preparation are shown in Fig. 10.

All glass samples, i.e. coated with coatings based on S1-S6, have very good anti-fog properties after preparation and storage at room temperature, as well as after storage in the refrigerator (3 °C) and freezer (− 20 °C).

The length of time that the finished preparations can be stored and will still retain very good anti-fog properties after application on glass was also tested. The same anti-fog tests were performed for glass samples treated with formulas on the day of preparation (day 1) and after aging, i.e. applied 3, 5, 10, 20, and 31 days after preparation. Preparations based on S1–S4 derivatives can be stored for at least a month and still retain their anti-fog properties after exposure glasses removed from room temperature, refrigerator (3 °C), and freezer (− 20 °C) to hot water. Preparations based on S6 had good anti-fog properties on the 10th day of the test (room temp., 3 °C and − 20 °C), and based on S5 had good anti-fog properties on the 10th day of the test (room temp.). Photos after formulas preparation and after 3, 5, 10, 20, and 31 days of aging are included in the Supplementary Information. Tests after storage of the samples in the refrigerator and freezer have shown the resistance of coatings (preservation of anti-fog properties) to low temperatures and sudden temperature changes.

After 2 months of storage in laboratory conditions (~ 22 °C, 35–45% RH) all coatings (applied after preparation the formulas) still retained their antifogging properties (photographs are included in the Supplementary Information).

**Figure 11:**
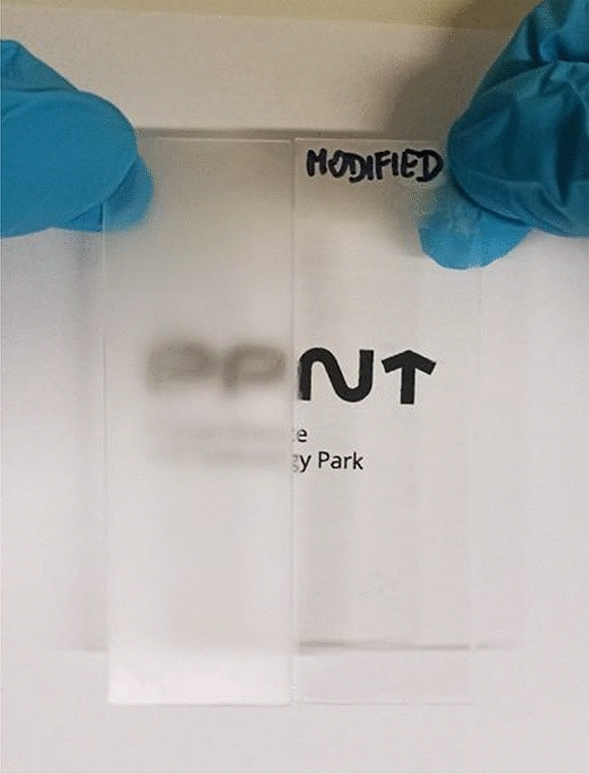
Antifogging test of glass uncoated (left side) and coated with derivative S4 (right side) when entering indoors (22 °C) from a cold outdoor environment (− 20 °C).

Next, an antifogging test was performed on the example of the S4 derivative, in which glass is removed from a low temperature (− 20 °C) and placed at room temperature (22 °C), as shown in Fig. 11. This test illustrates a situation where a person wearing eyeglasses enters a warm room (22 °C) from a cold outdoor environment (− 20 °C).

In this situation, the uncoated glass slide (left side) immediately became opaque, while the coated one (right side) remained clear, demonstrating the antifogging performance of coating (S4).

**Figure 12:**
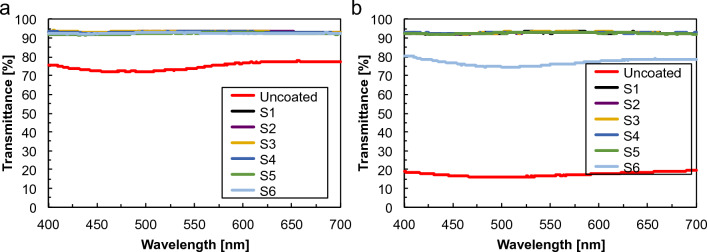
Light transmission measured after (**a**) 15 s exposure to hot water vapor (5 cm above 80 °C water) and (**b**) after 30 min storage in a freezer at -20 °C, immediately (~ 15 s) after samples transfer to ambient lab conditions (temperature 22 °C, 38% RH).

To perform the antifogging test quantitively, all samples were held 5 cm above hot water (80 °C, RH = 100%) for 15 s, followed by moving the samples at ambient lab conditions (22 °C, 38% RH) and the light transmittance in the range of 400–700 nm was recorded on UV–vis spectrophotometer (Fig. 12).

The light transmittance values of the uncoated (~ 90.5%) and coated (~ 92.5%) glass slides are comparable before the test (Fig. 5b). After the hot-vapor test, the light transmittance of the uncoated glass slide was reduced to ~ 75%, whereas all coated samples still retained high light transmission (~ 92.5%) (Fig. 12a). These results indicated that all coated surfaces can render optically clear surfaces and have excellent antifogging properties. To evaluate the frost resistance of the coatings quantitatively, the transmittance of samples held in the freezer at − 20 °C for 30 min (Fig. 12b), followed by moving the samples at ambient lab conditions (22 °C, 38% RH) and the light transmittance was recorded. Light transmittance of uncoated glass slide decreased to ~ 17.5%. The S1–S5 coated samples remained optically clear and frost-free to show excellent frost resistance maintaining a high transmittance (∼92.5%). Only the light transmission value of S6 slightly decreased to about 77%, however, it is still at a high level. Lower clarity and transmission of the S6 coating may result from its stronger water absorbing capacity. The excessively absorbed water in the coating may deteriorate the frost-resisting properties.

**Figure 13:**
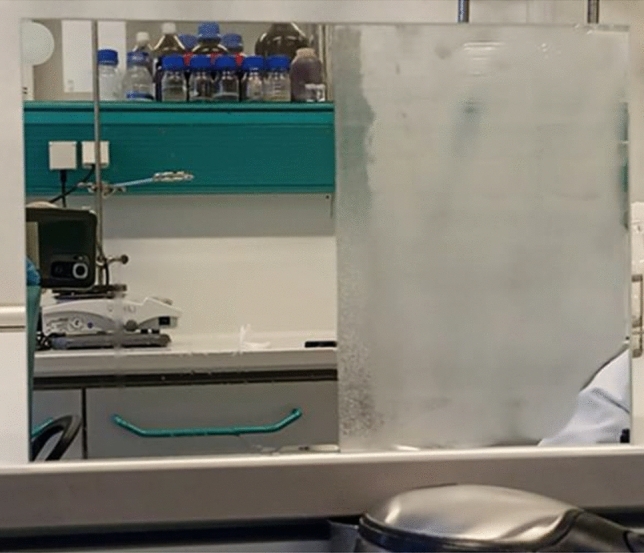
Photograph of uncoated (right side) and coated with derivative S4 (left side) mirror under the antifogging test.

During the last test, the surface of a glass mirror was exposed to hot water vapor, as shown in Fig. 13.

The right side of the mirror was raw, while the left side was coated with a preparation based on the S4 derivative. It was observed that the surface of the coated mirror was not fogged, indicating the excellent antifogging ability of the coatings. The video available in the Supplementary Information shows the real-time observation of the excellent antifogging property of the coating upon exposure to water vapor.

The preparation based on S4 was stored for 8 months in a laboratory environment, after which the glass plate was coated with it and placed 1.5 cm above a hot (~ 65 °C) bath (photo in Supplementary Information). The glass covered with S4 (after 8 months of aging) shows long-term durability and stability of preparation and is promising for real-world applications.

## Conclusions

Six silanes containing an alkoxysilyl group and a polyether chain were obtained as a result of a one-step, quick, and simple thiol-ene click reaction. Thanks to the presence of the alkoxysilyl group, bonding of compounds to the glass surface was possible, while the presence of the polyether group ensured (super)hydrophilicity and anti-fogging properties. The application of the coatings on the surfaces of the glass samples was confirmed by FT-IR spectroscopy. All modified glass slides are characterized by excellent optical clarity. The measured values of WCAs showed superhydrophilic or hydrophilic properties of all modified samples. All samples retained their superhydrophilic or hydrophilic properties during storage for at least a month in ambient lab conditions (~ 22 °C and 35–45% humidity). All samples retained their antifogging properties during storage for at least 2 months in laboratory conditions. WCA values do not correlate with anti-fog properties. In two cases (derivatives S5 and S6), the WCA values of the modified samples were higher than those of the raw glass, but they were characterized by very good anti-fog properties. Moreover, a decrease in the WCAs values was observed during their storage, however, the anti-fog properties deteriorated during this time. After 3 weeks of storage at 60 °C and − 20 °C, the glass samples had superhydrophilic or hydrophilic properties. A difference in the results of contact angles was noted for the S5 and S6 derivatives that have an additional alkyl linker in the structure. For derivatives S5 and S6 the contact angles decreased over time of storage, while the anti-fog properties deteriorated. Differences were observed in WCA after storage the glass at 60 °C depending on the type of terminal groups, i.e. OH versus OCH_3_. For glass samples covered with derivatives containing OH terminal groups, a significantly higher increase in WCAs values over time was observed than for glass samples coated with derivatives containing the OCH_3_ terminal group. Coatings S1–S5 have excellent frost-resisting properties.

### Supplementary Information

Supplementary Information 1.

Supplementary Video 1.

## Data Availability

The datasets generated during the current study are available from the corresponding author on reasonable request.
