# Supplementary material for: Thiol-ene click reaction as an effective tool for the synthesis of PEG-functionalized alkoxysilanes-precursors of anti-fog coatings
Source: Sci Rep. 2023 Nov 29;13:21025. doi: 10.1038/s41598-023-48192-4 (PMC10687060; doi:10.1038/s41598-023-48192-4)
Supplement: Supplementary file 1 — Supplementary Information 1. [file 41598_2023_48192_MOESM1_ESM.pdf]

SUPPLEMENTARY INFORMATION

Thiol-ene click reaction as an effective tool for the synthesis of PEG-functionalized alkoxysilanes - precursors of anti-fog coatings

Marta Kaczmarek[1], Agnieszka Przybylska[1], Anna Szymańska[2,*], Agnieszka Dutkiewicz[2], and Hieronim Maciejewski[1,2]

[1]Faculty of Chemistry, Adam Mickiewicz University, Uniwersytetu Poznańskiego 8, 61-614 Poznań, Poland

[2]Poznań Science and Technology Park, Adam Mickiewicz University Foundation, Rubież 46, 61-612 Poznań, Poland

[*]anna.szymanska@ppnt.poznan.pl

Table of contents

1. Products characterization ... 3
1.1 S1 ... 3
Product characterization ... 3
NMR spectra ... 3
FT-IR spectrum ... 5
1.2 S2 ... 5
Product characterization ... 5
NMR spectra ... 6
FT-IR spectrum ... 7
1.3 S3 ... 8
Product characterization ... 8
NMR spectra ... 8
FT-IR spectrum ... 10
1.4 S4 ... 10
Product characterization ... 10
NMR spectra ... 11
FT-IR spectrum ... 12
1.5 S5 ... 13
Product characterization ... 13
NMR spectra ... 13
FT-IR spectrum ... 15
1.6 S6 ... 15
Product characterization ... 15
NMR spectra ... 15
FT-IR spectrum ... 17
2. Photographs of glass slides over hot steaming water after the modification (1 day) ... 18
3. Photographs of glass slides over hot steaming water after 3 days of aging the solutions ... 19
4. Photographs of glass slides over hot steaming water after 5 days of aging the solutions ... 20
5. Photographs of glass slides over hot steaming water after 10 days of aging the solutions ... 21
6. Photographs of glass slides over hot steaming water after 20 days of aging the solutions ... 22
7. Photographs of glass slides over hot steaming water after 31 days of aging the solutions ... 24
8. Photograph of glass slide over hot steaming water after 8 months of aging S4 solution ... 25
9. WCAs values of coated samples measured after the modification (1 day) and after storage the solutions in a laboratory environment ... 25
10. WCAs values of coated samples measured after storage at 60 °C ... 25
11. WCAs values of coated samples measured after storage at -20 °C ... 25
12. Photographs of glass slides over hot steaming water after 2 months of glass storage ... 26
13. Materials of the antifogging coatings ... 26

1. Products characterization

1.1 S1

Product characterization

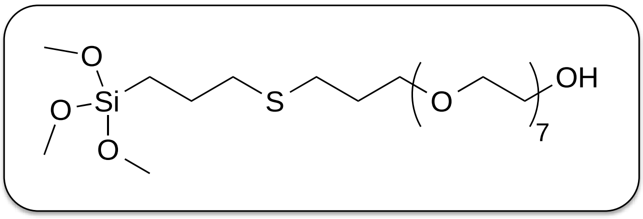

^{1}H NMR (400 MHz, $CDCl_3$): δ 3.65 (m, 2H, CH_2OH); 3.57 (m, 26H, CH_2O); 3.51 (m, 9H, $Si(OCH_3)_3$); 3.47 (d, J = 6.3 Hz, 2H, $OC\underline{H}_2CH_2CH_2S$); 3.38 (s, 1H, OH); 2.48 (dtd, J = 10.6, 7.4, 3.0 Hz, 4H, CH_2SCH_2); 1.78 (m, 2H, $SCH_2C\underline{H}_2$); 1.63 (m, 2H, $SiCH_2C\underline{H}_2$); 0.69 (t, 2H, $SiCH_2$) ppm. **^{13}C NMR (101 MHz, $CDCl_3$):** δ 72.52 (CH_2O); 70.58 (CH_2O); 70.52(CH_2O); 70.29(CH_2O); 70.15(CH_2O); 69.75 (CH_2O); 61.64 (CH_2OH); 50.48 ($Si(OCH_3)_3$); 34.98 ($SiCH_2CH_2\underline{C}H_2S$); 29.66 ($SCH_2CH_2$); 28.51($SCH_2$); 22.91($SiCH_2CH_2$); 8.53 ($SiCH_2$) ppm. **^{29}Si NMR** (79 MHz, $CDCl_3$): δ -41,76 ($Si(OCH_3)_3$) ppm.

FT-IR (ATR, cm^{-1}): 3490, 2885, 2841, 1455, 1349, 1295, 1250, 1189, 1078, 949, 809.

NMR spectra

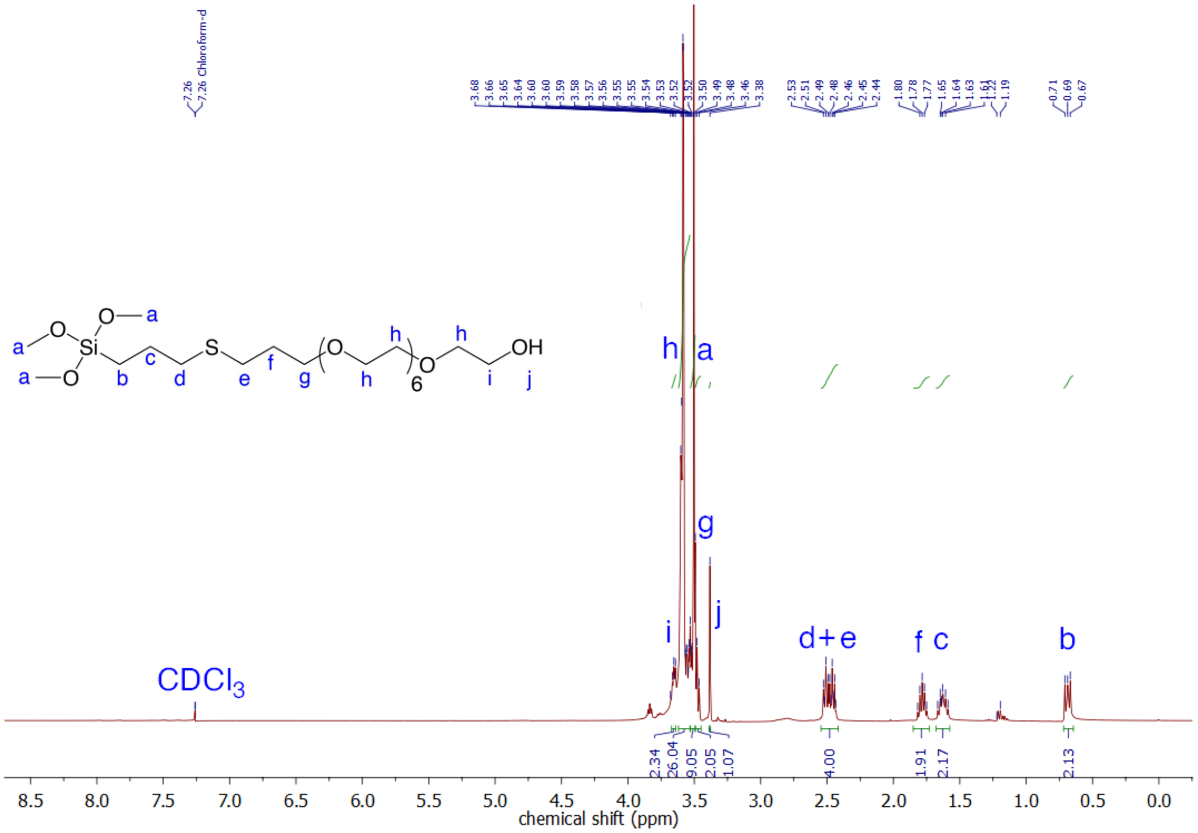

Figure S1. ^{1}H NMR spectrum of S1

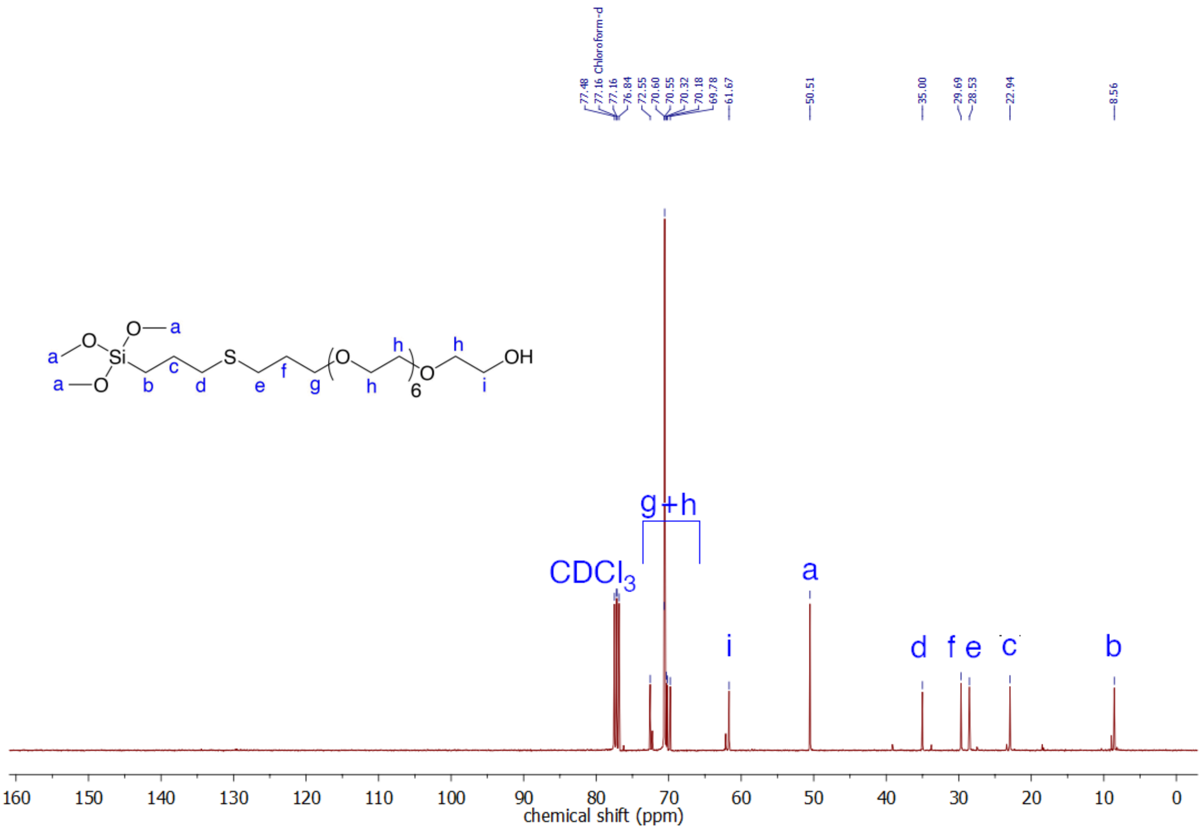

Figure S2. ^{13}C NMR spectrum of S1

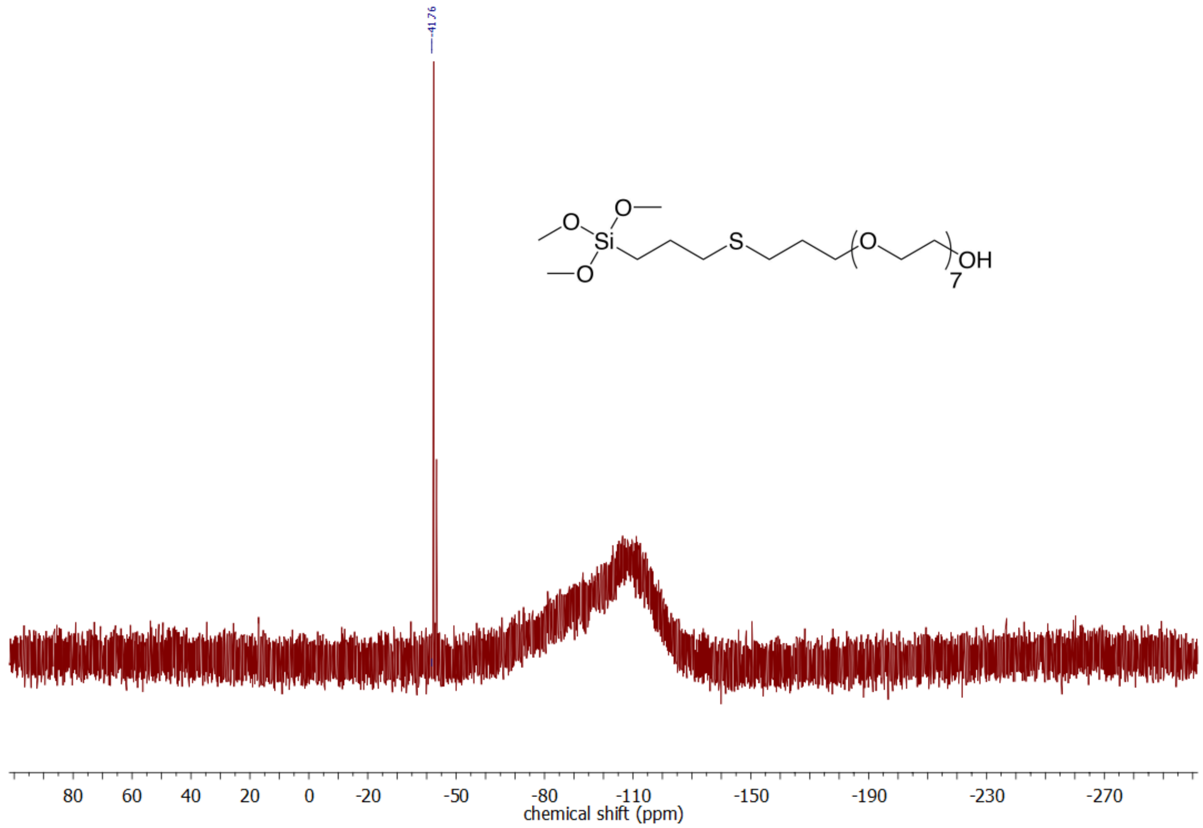

Figure S3. ^{29}Si NMR spectrum of S1

FT-IR spectrum

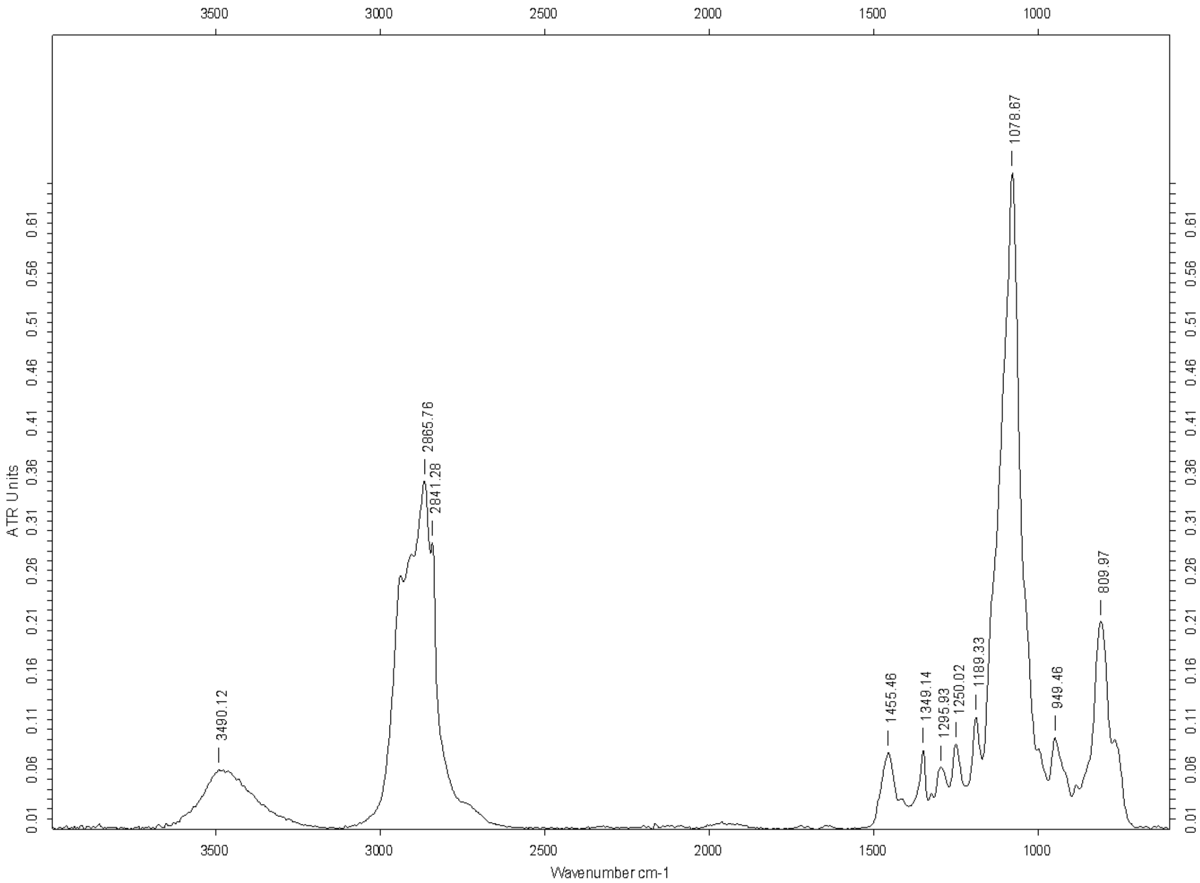

Figure S4. FT-IR spectrum of S1

1.2 S2

Product characterization

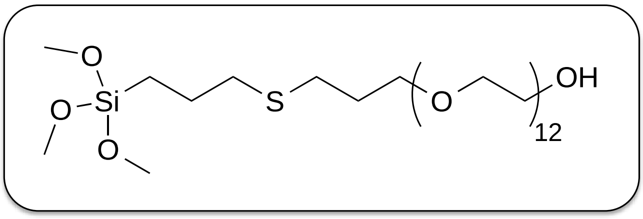

^{1}H NMR (400 MHz, $CDCl_3$): δ 3.65 (s, 2H, CH_2OH); 3.56 (m, 46H, CH_2O); 3.50 (m, 9H, $Si(OCH_3)_3$); 3.47 (d, *J* = 6.3 Hz, 2H, $SCH_2CH_2C\underline{H}_2O$); 3.38 (s, 1H, OH); 2.48 (dt, *J* = 19.2, 7.3 Hz, 4H, CH_2SCH_2); 1.77 (dd, *J* = 13.8, 6.9 Hz, 2H, $SCH_2C\underline{H}_2$); 1.63 (ddd, *J* = 15.5, 10.6, 7.6 Hz, 2H, $SiCH_2C\underline{H}_2$); 0.69 (t, 2H, $SiCH_2$) ppm. **^{13}C NMR (101 MHz, $CDCl_3$):** δ 71.90 (CH_2O); 70.54(CH_2O); 70.48 (CH_2O); 70.17 (CH_2O); 69.77(CH_2O); 58.99 (OCH_3); 50.50 ($Si(OCH_3)_3$); 34.99 ($SiCH_2CH_2\underline{C}H_2S$); 29.68 ($SCH_2$); 28.52 ($SCH_2CH_2$); 22.93($SiCH_2CH_2$); 8.55 ($SiCH_2$) ppm. **^{29}Si NMR (79 MHz, $CDCl_3$):** δ -42.27 $Si(OCH_3)_3$) ppm.

FT-IR (ATR, cm^{-1}): 3479, 2864, 1455, 1349, 1296, 1249, 1189, 1080, 948, 812.

NMR spectra

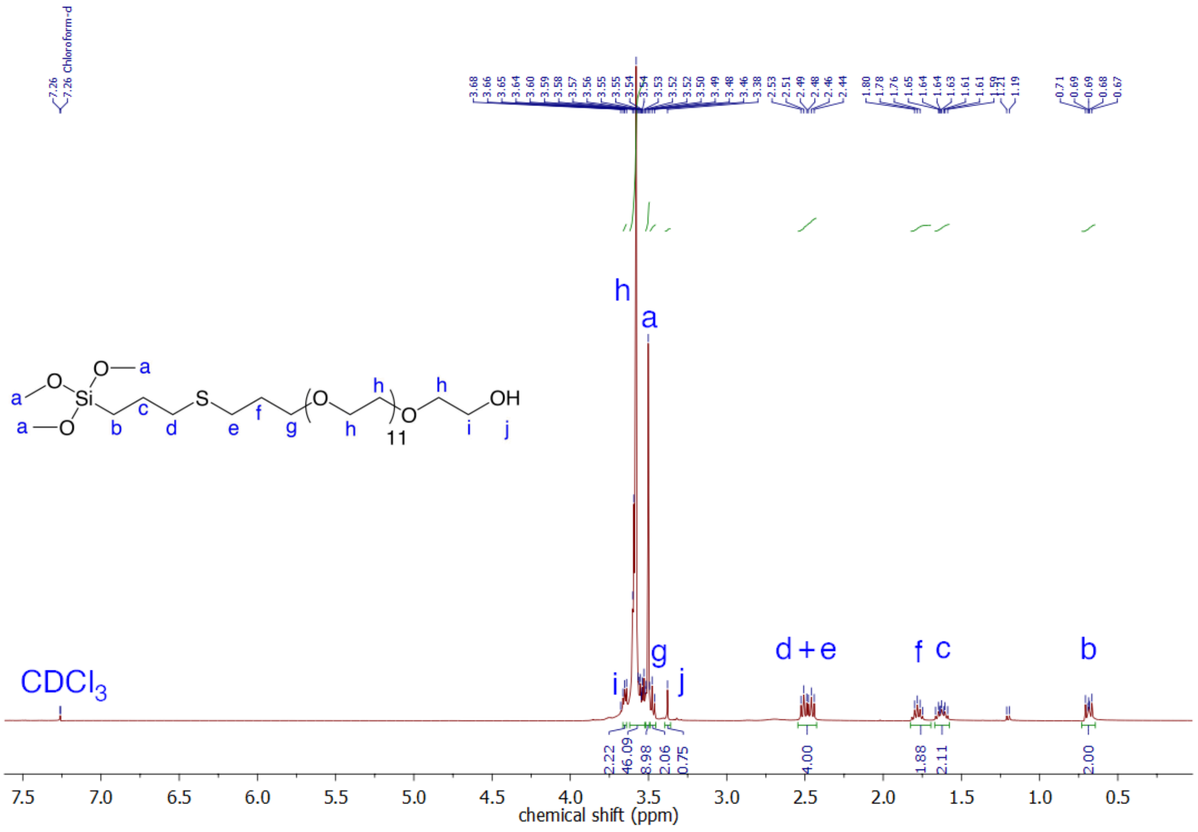

Figure S5. ^{1}H NMR spectrum of S2

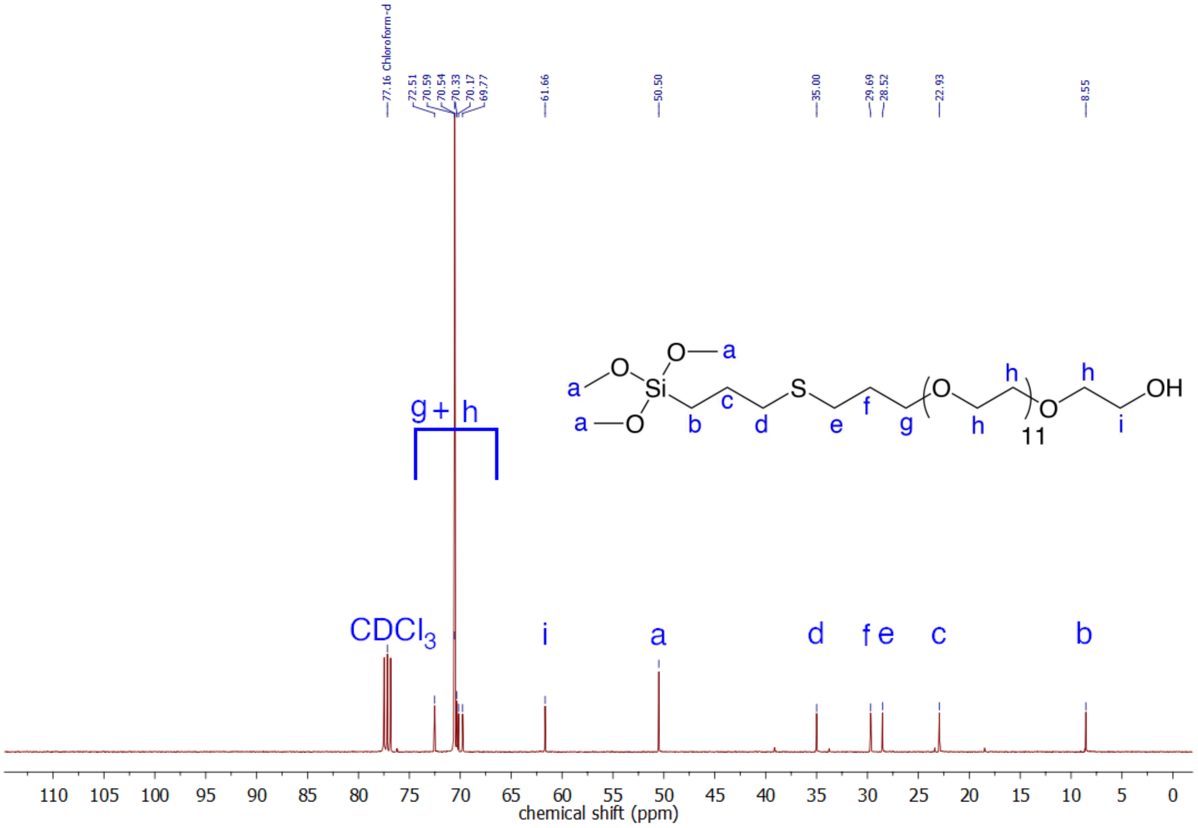

Figure S6. ^{13}C NMR spectrum of S2

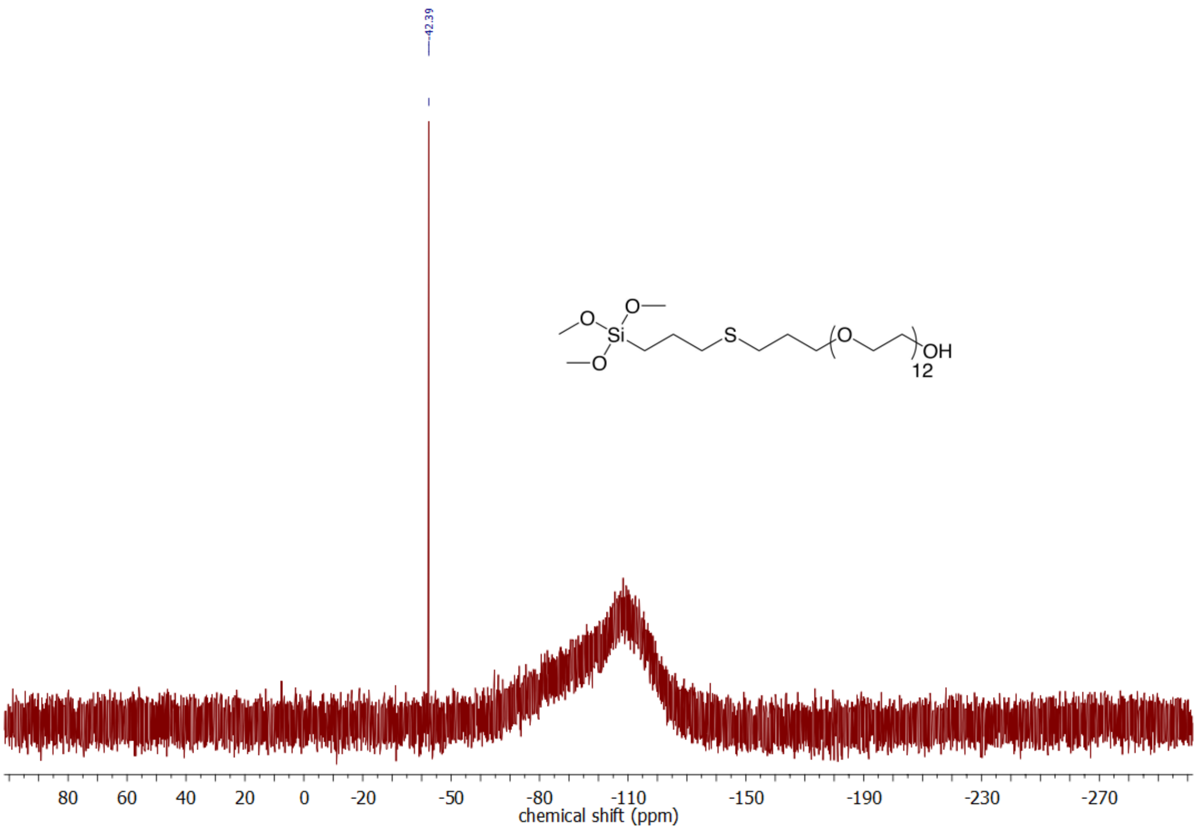

Figure S7. ^{29}Si NMR spectrum of S2

FT-IR spectrum

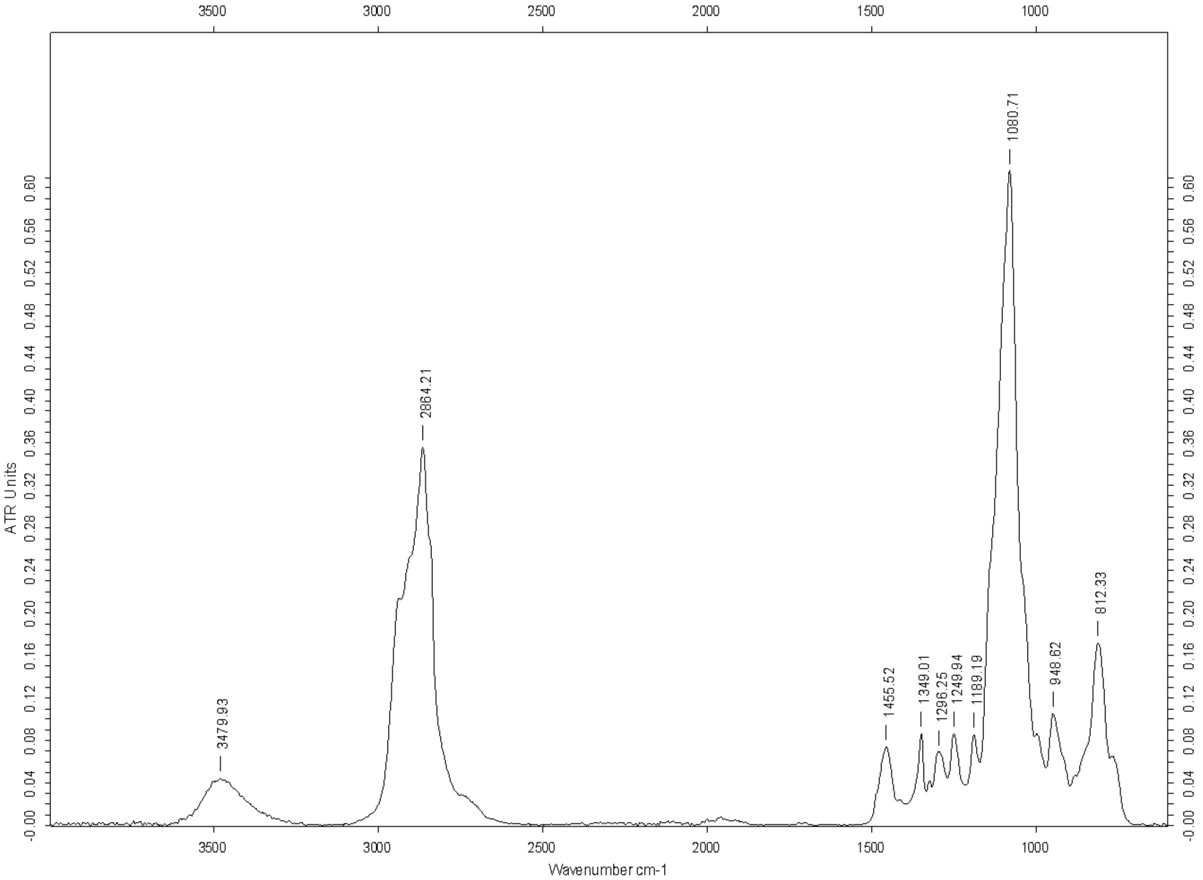

Figure S8. FT-IR spectrum of S2

1.3 S3

Product characterization

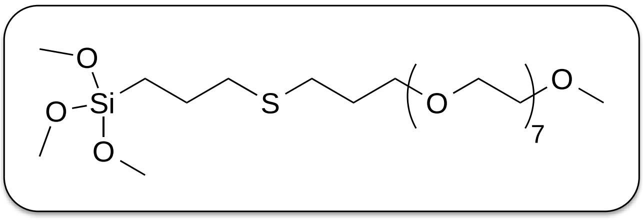

^{1}H NMR (400 MHz, $CDCl_3$): δ 3.57 (m, 28H, CH_2O); 3.52 (m, 9H, $Si(OCH_3)_3$); 3.49 (m, 2H, $SCH_2CH_2CH_2$); 3.33 (s, 3H, OCH_3); 2.50 (m, 4H, CH_2SCH_2); 1.80 (m, 2H, SCH_2CH_2); 1.65 (dt, J = 15.7, 7.6 Hz, 2H, $SiCH_2C\underline{H}_2$); 0.71 (dd, J = 9.4, 7.1 Hz, 2H, $SiCH_2$) ppm. **^{13}C NMR (101 MHz, $CDCl_3$):** δ 71.95 (CH_2O); 70.62(CH_2O); 70.59(CH_2O); 70.53 (CH_2O); 70.22 (CH_2O); 69.82 (CH_2O); 59.04 (OCH_3); 50.54 ($Si(OCH_3)_3$); 35.05 ($SiCH_2CH_2\underline{C}H_2S$); 29.73 ($SCH_2CH_2$); 28.58 ($SCH_2$); 22.97($SiCH_2CH_2$); 8.59 ($SiCH_2$) ppm. **^{29}Si NMR (79 MHz, $CDCl_3$):** δ -42.28 ($Si(OCH_3)_3$) ppm.
FT-IR (ATR, cm^{-1}): 2865, 2840, 1455, 1349, 1296, 1249, 1190, 1080, 950, 810.

NMR spectra

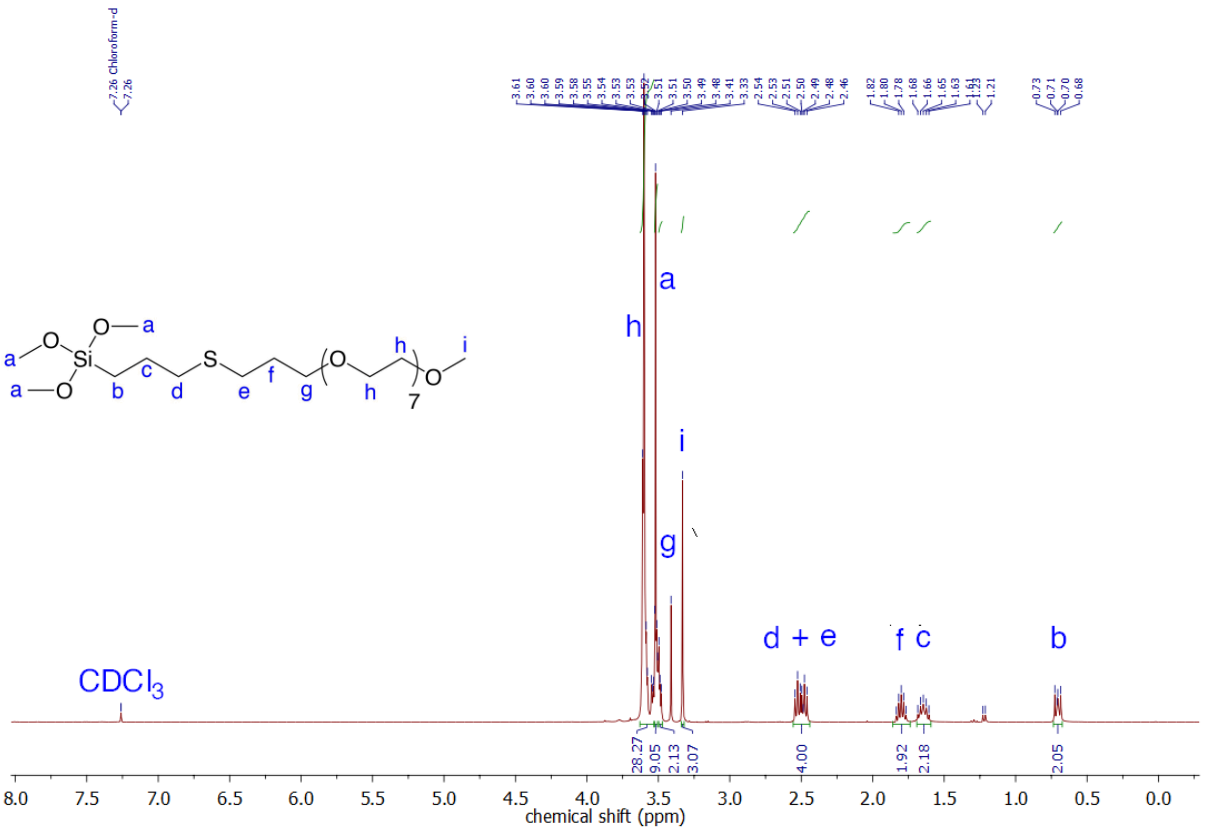

Figure S9. ^{1}H NMR spectrum of S3

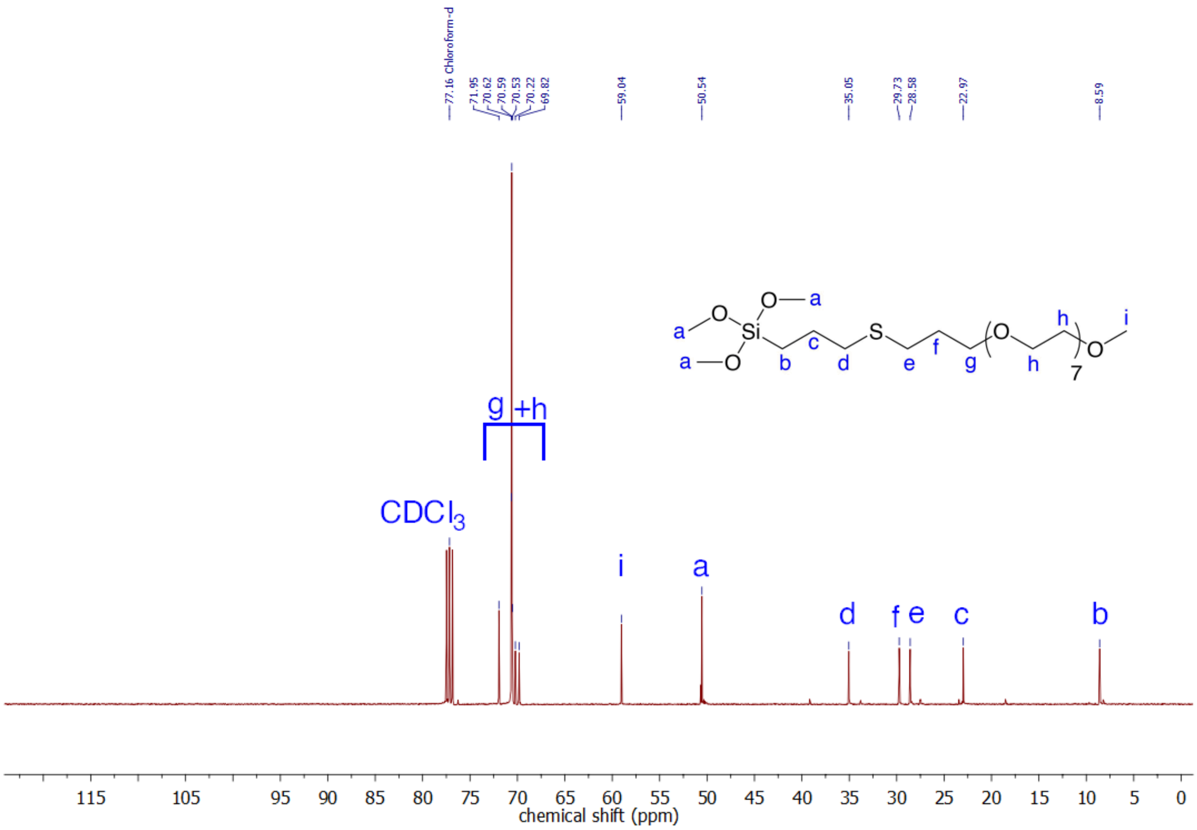

Figure S10. ^{13}C NMR spectrum of S3

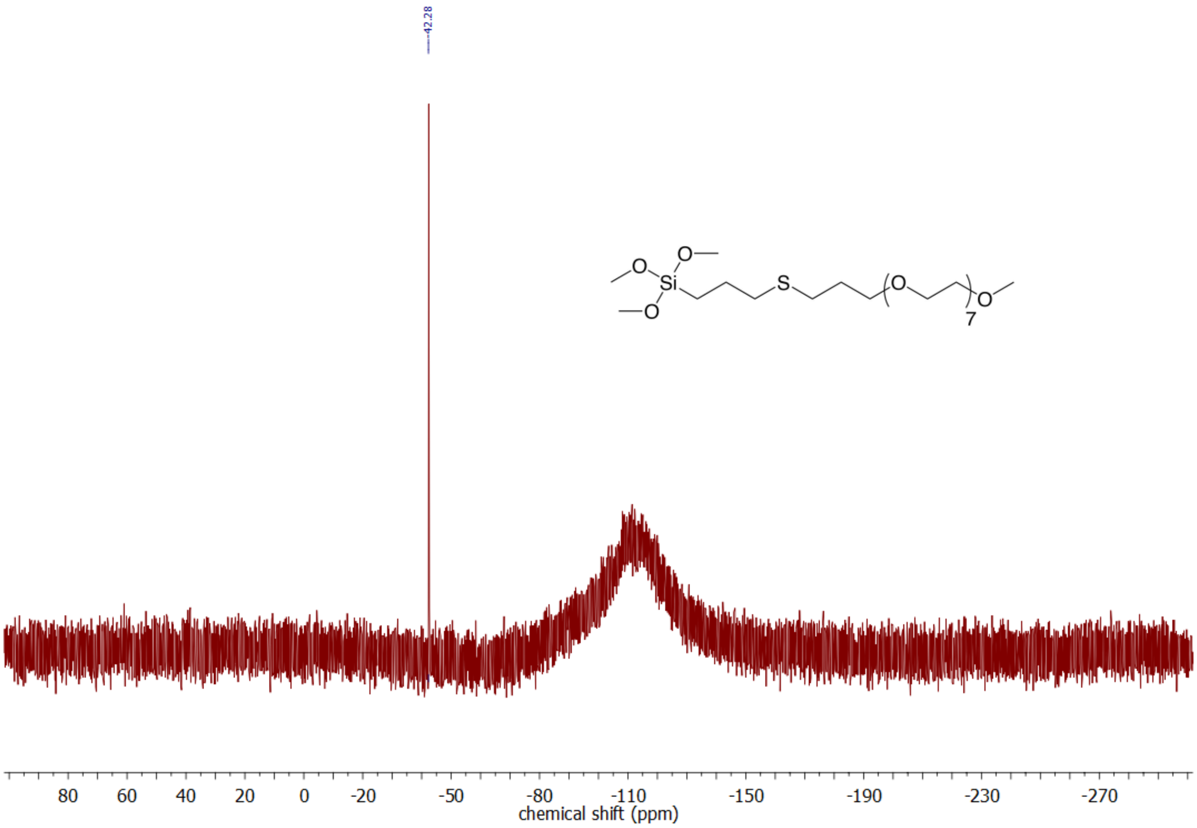

Figure S11. ^{29}Si NMR spectrum of S3

FT-IR spectrum

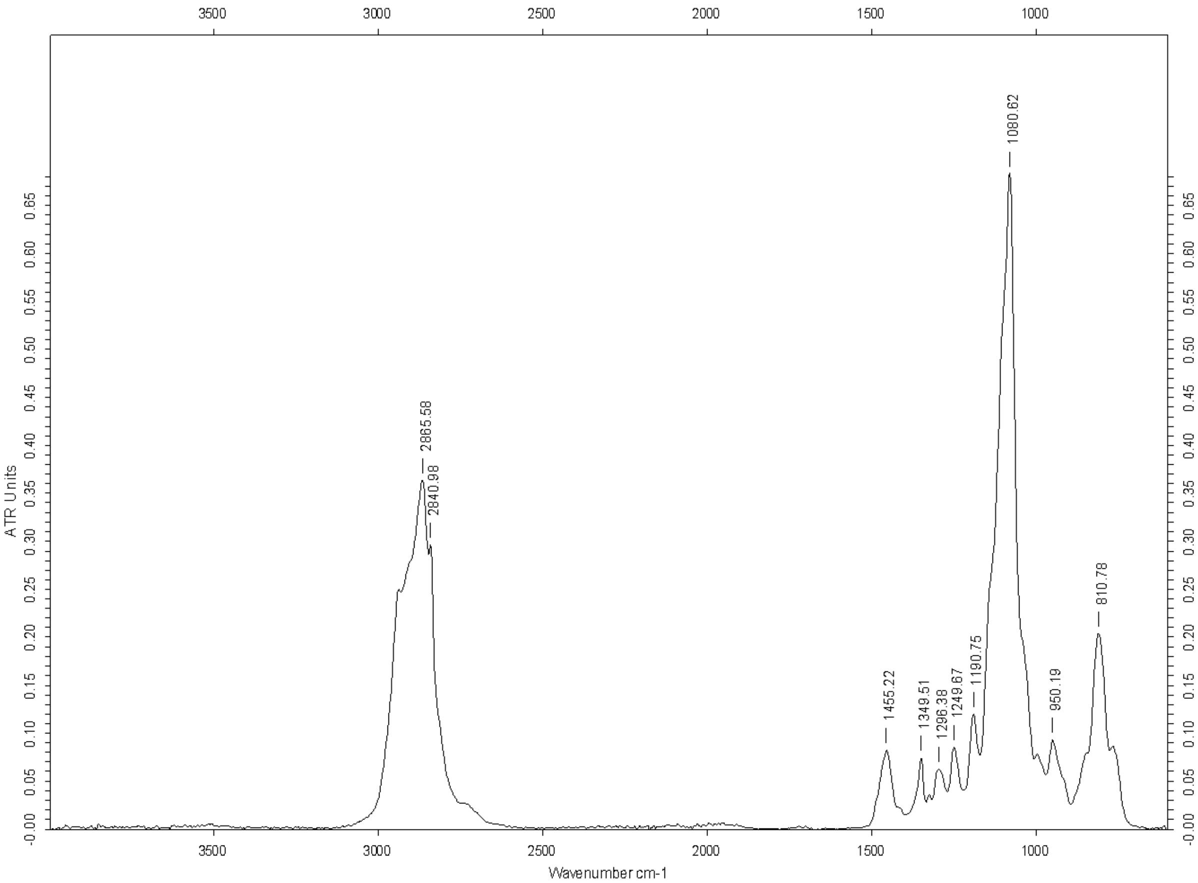

Figure S12. FT-IR spectrum of S3

1.4 S4

Product characterization

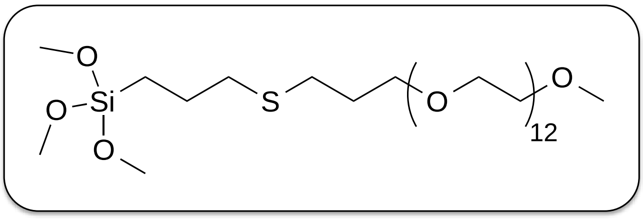

^{1}H NMR (400 MHz, $CDCl_3$): δ 3.57 (m, 48H, CH_2O); 3.51 (m, 9H, $Si(OCH_3)_3$); 3.48 (m, 2H, $SCH_2CH_2CH_2$); 3.32 (s, 3H, OCH_3); 2.49 (dt, *J* = 19.2, 7.3 Hz, 4H, CH_2SCH_2); 1.79 (m, 2H, $SCH_2C\underline{H}_2$); 1.63 (m, 2H, $SiCH_2C\underline{H}_2$); 0.69 (dd, *J* = 9.4, 7.1 Hz, 2H, $SiCH_2$) ppm. **^{13}C NMR (101 MHz, $CDCl_3$):** δ 71.90 (CH_2O); 70.54(CH_2O); 70.48 (CH_2O); 70.17 (CH_2O); 69.77(CH_2O); 58.99 (OCH_3); 50.50 ($Si(OCH_3)_3$); 34.99 ($SiCH_2CH_2\underline{C}H_2S$); 29.68 ($SCH_2$); 28.52 ($SCH_2CH_2$); 22.93 ($SiCH_2CH_2$); 8.55($SiCH_2$) ppm. **^{29}Si NMR (79 MHz, $CDCl_3$):** δ -42.27 $Si(OCH_3)_3$) ppm.

FT-IR (ATR, cm^{-1}): 2865, 1455, 1349, 1324, 1296, 1249, 1190, 1082, 996, 949, 812.

NMR spectra

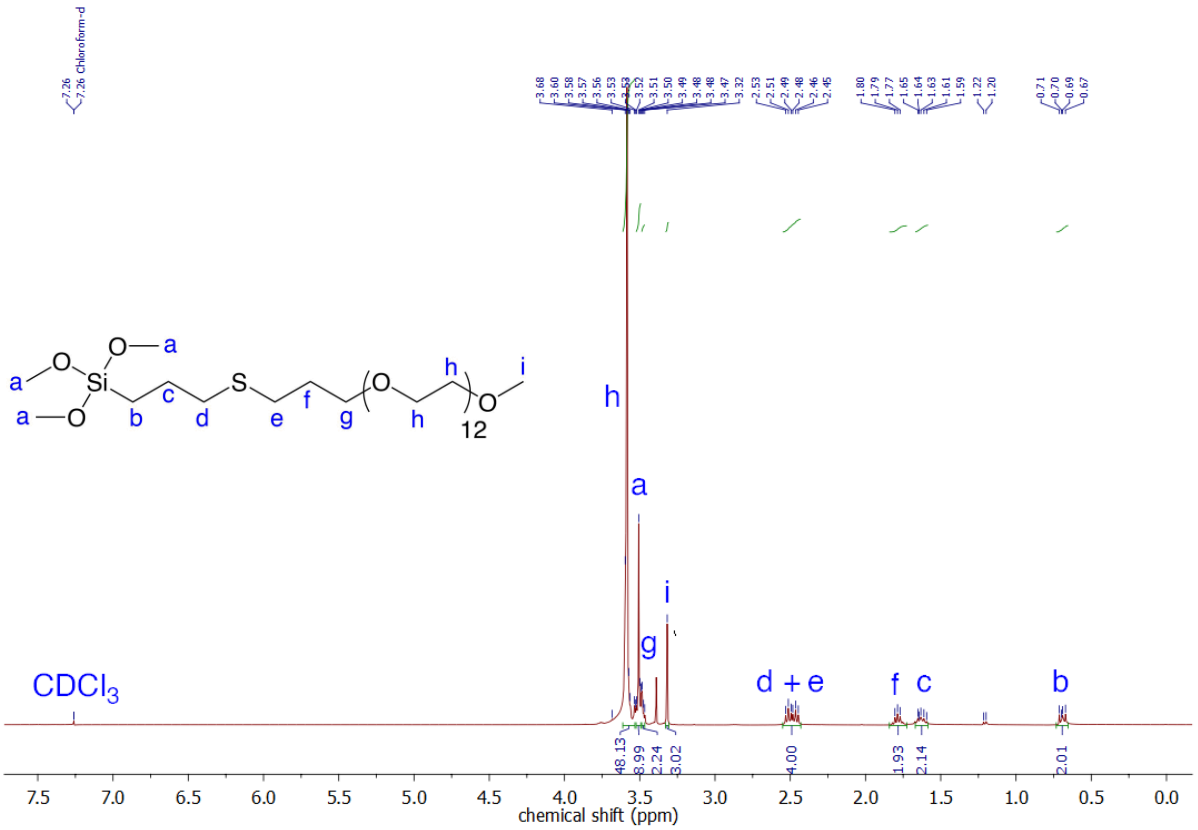

Figure S13. ^{1}H NMR spectrum of S4

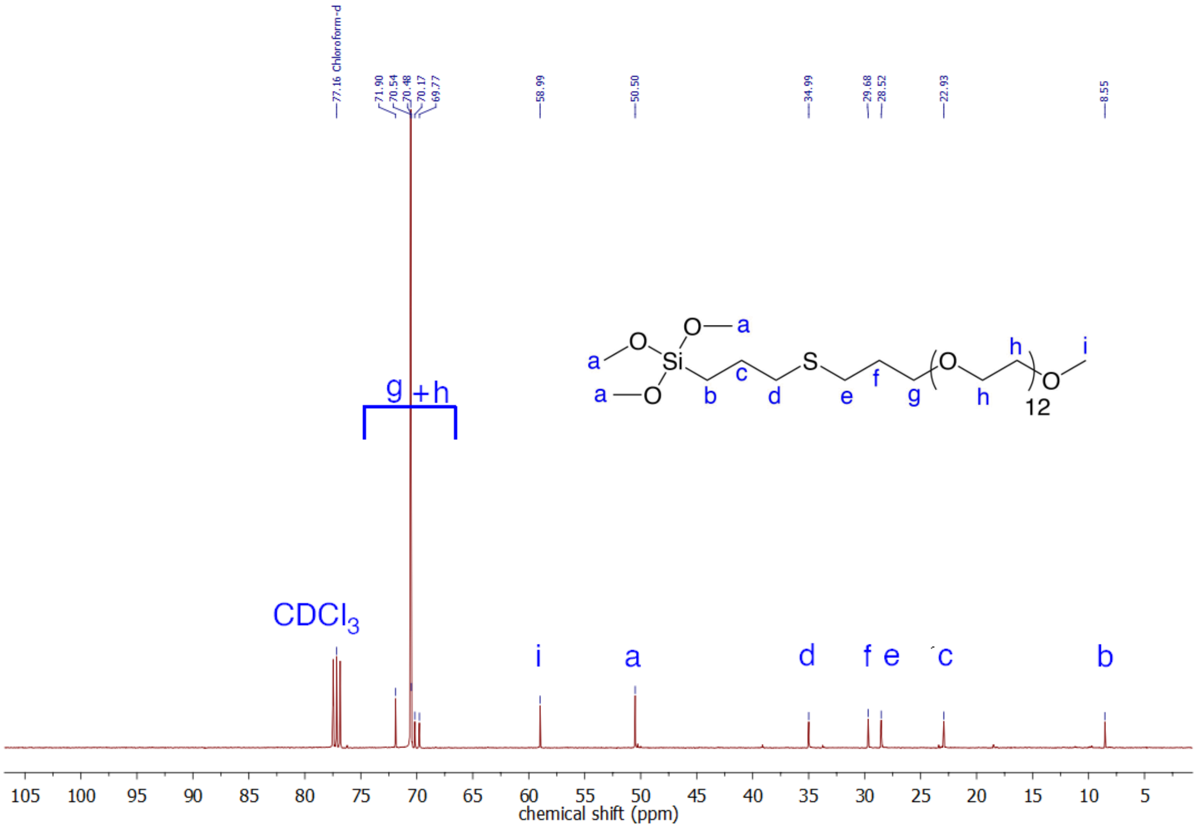

Figure S14. ^{13}C NMR spectrum of S4

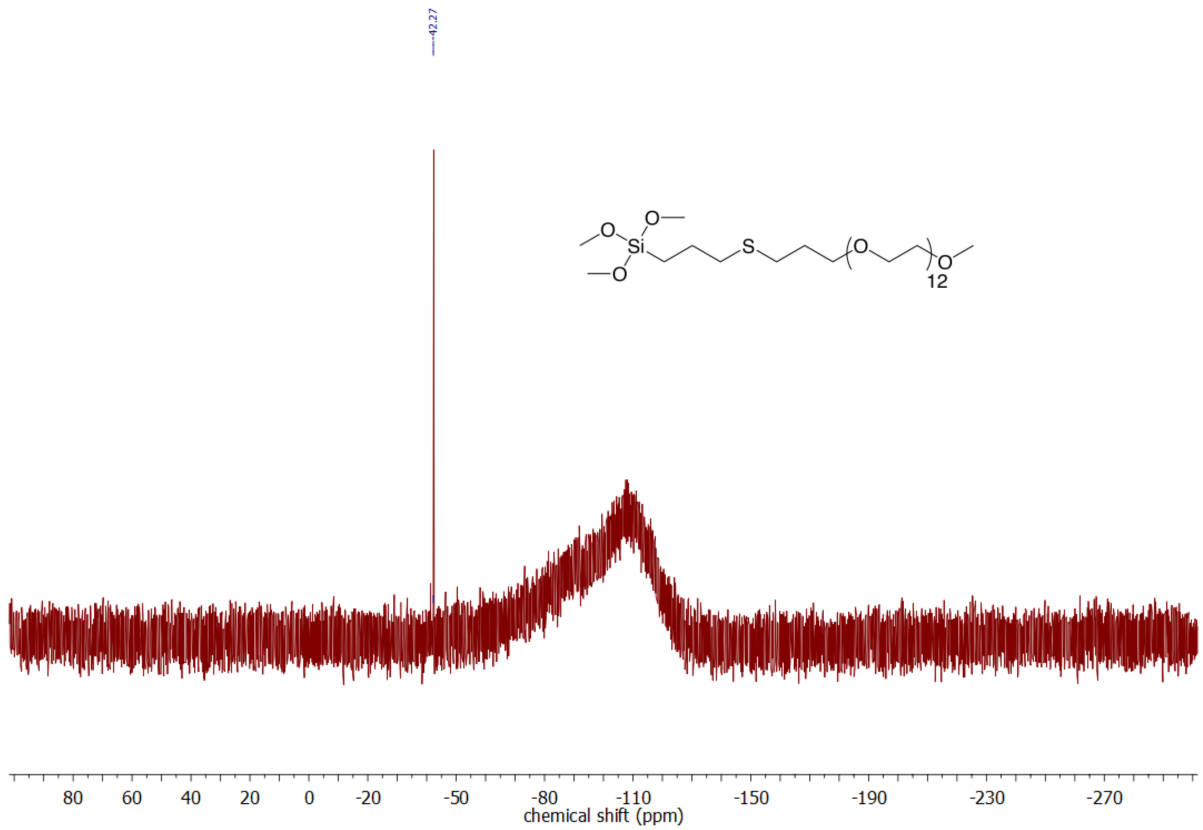

Figure S15. ^{29}Si NMR spectrum of S4

FT-IR spectrum

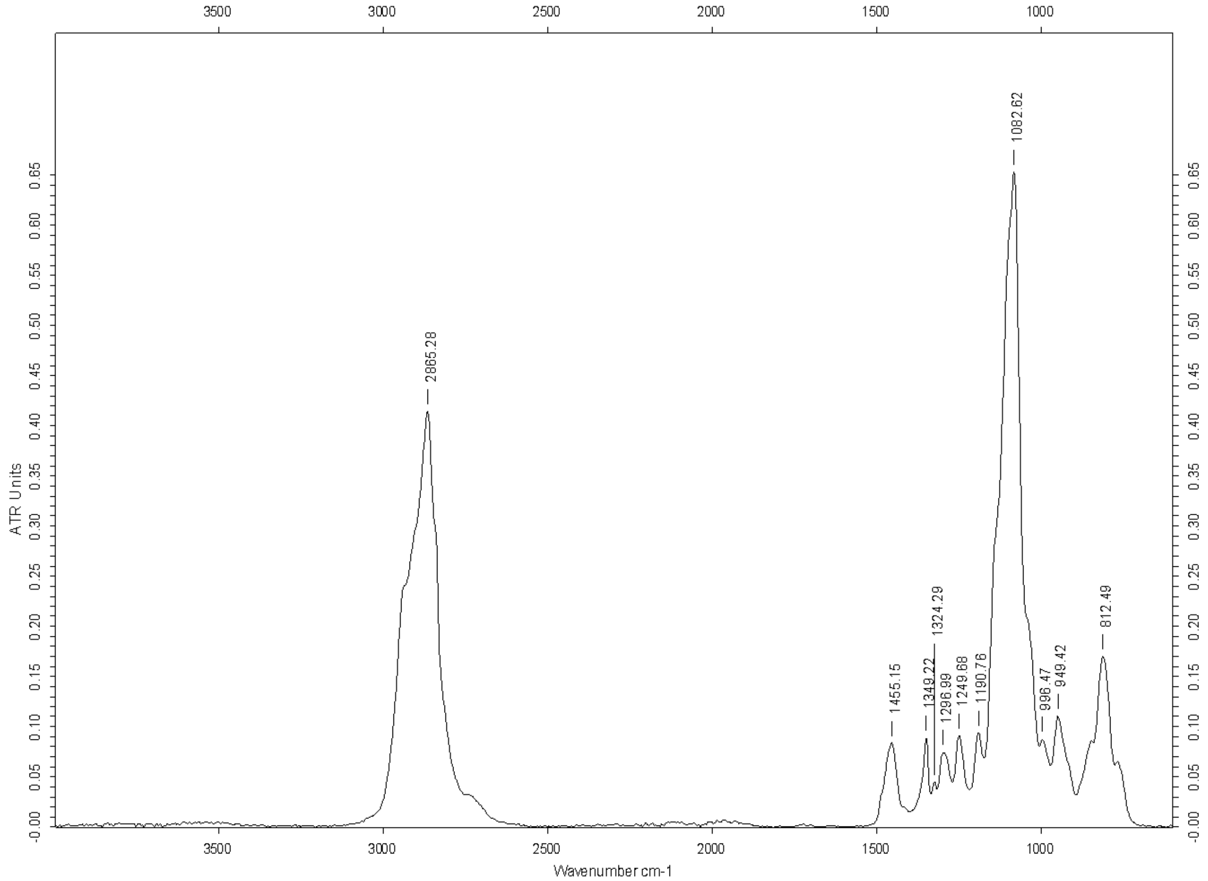

Figure S16. FT-IR spectrum of S4

1.5 S5

Product characterization

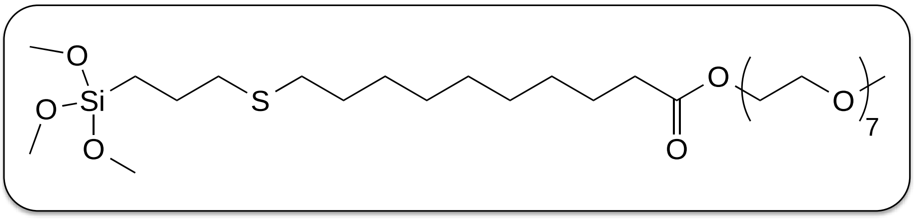

^{1}H NMR (400 MHz, $CDCl_3$): δ 4.19 (m, 2H, $C(O)OCH_2$); 3.63 (m, 26H, CH_2O); 3.53 (m, 9H, $SiOCH_3$); 3.34 (s, 3H, OCH_3); 2.49 (m, 4H, CH_2SCH_2); 2.28 (t, J = 7.6 Hz, 2H, $CH_2C(O)$); 1.71-54 (dtd, J = 27.7, 15.2, 7.7 Hz, 6H, $SiCH_2C\underline{H}_2$, $C\underline{H}_2$, $SCH_2C\underline{H}_2$); 1.27 (m, 10H, CH_2); 0.72 (t, 2H, $SiCH_2$) ppm. **^{13}C NMR (101 MHz, $CDCl_3$):** δ 173.77 (C=O); 71.94 ($\underline{CH}_2OCH_3$); 70.59 (CH_2O); 69.20 ($C(O)OCH_2\underline{C}H_2$); 63.36 ($C(O)O\underline{C}H_2$); 59.02 ($OCH_3$); 50.53 ($Si(OCH_3)_3$); 35.07 ($SiCH_2CH_2\underline{C}H_2S$); 34.18 ($C(O)CH_2$); 31.96, 29.73, 29.34, 29.21, 29.20, 29.10, 28.90 (CH_2), 24.89 ($C(O)CH_2\underline{C}H_2$); 23.01 ($SiCH_2\underline{C}H_2$); 8.59 ($SiCH_2$) ppm. **^{29}Si NMR (79 MHz, $CDCl_3$):** δ -42.31 ($Si(OCH_3)$) ppm.

FT-IR (ATR, cm^{-1}): 3512, 2925, 2857, 1734, 1456, 1346, 1297, 1249, 1188, 1081, 950, 811.

NMR spectra

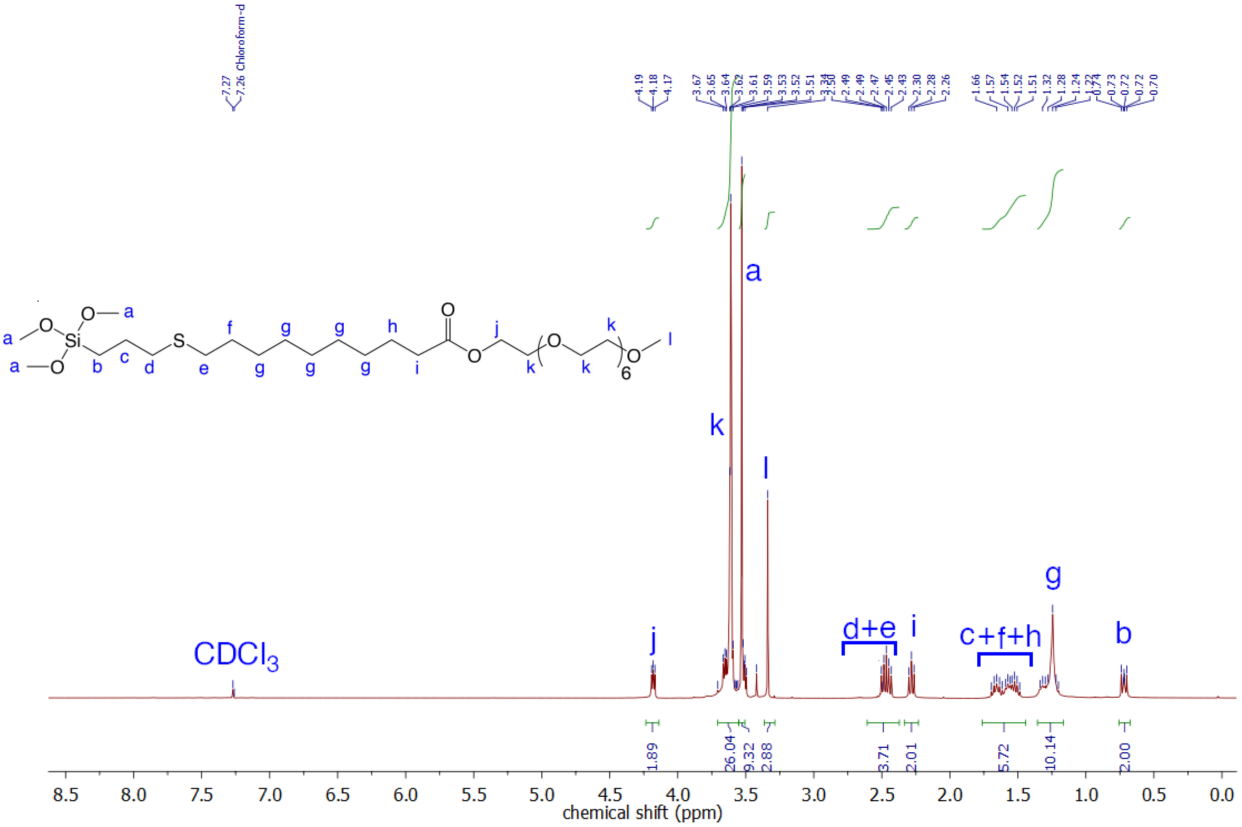

Figure S17. ^{1}H NMR spectrum of S5

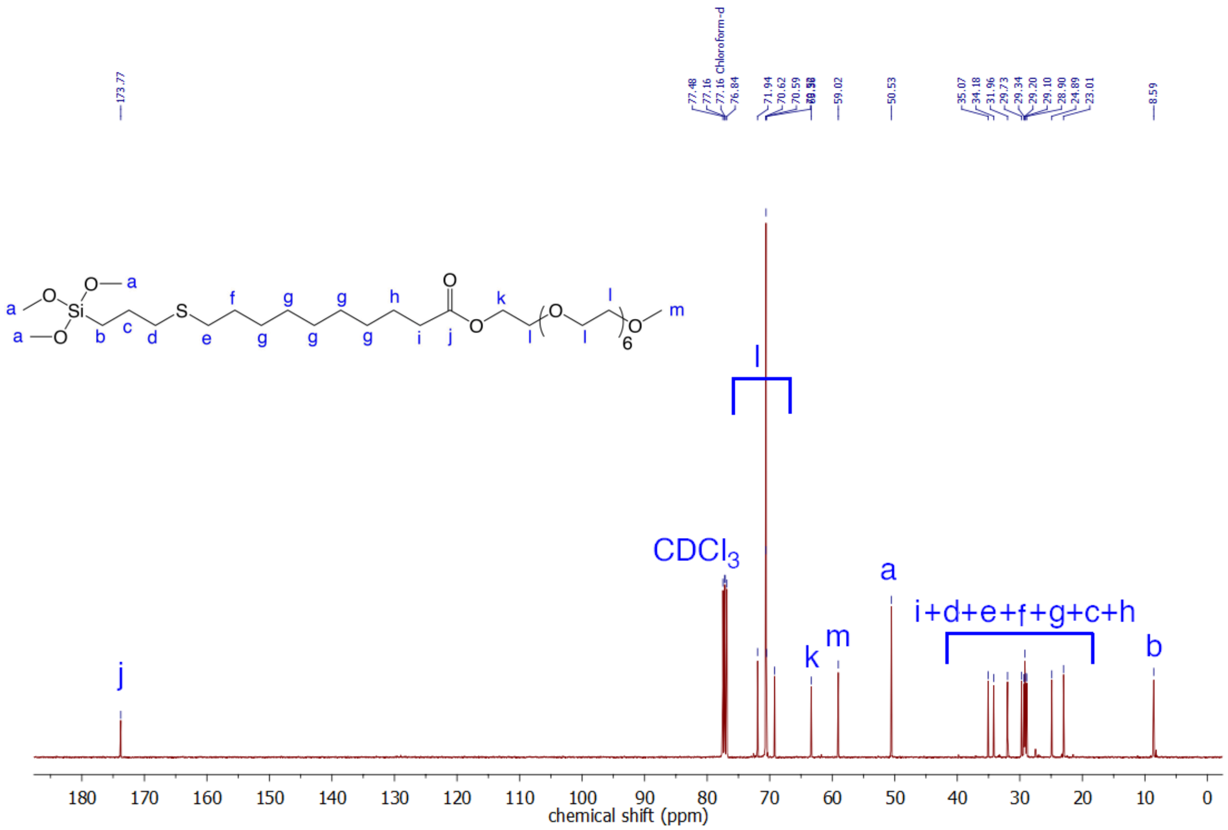

Figure S18. ^{13}C NMR spectrum of S5

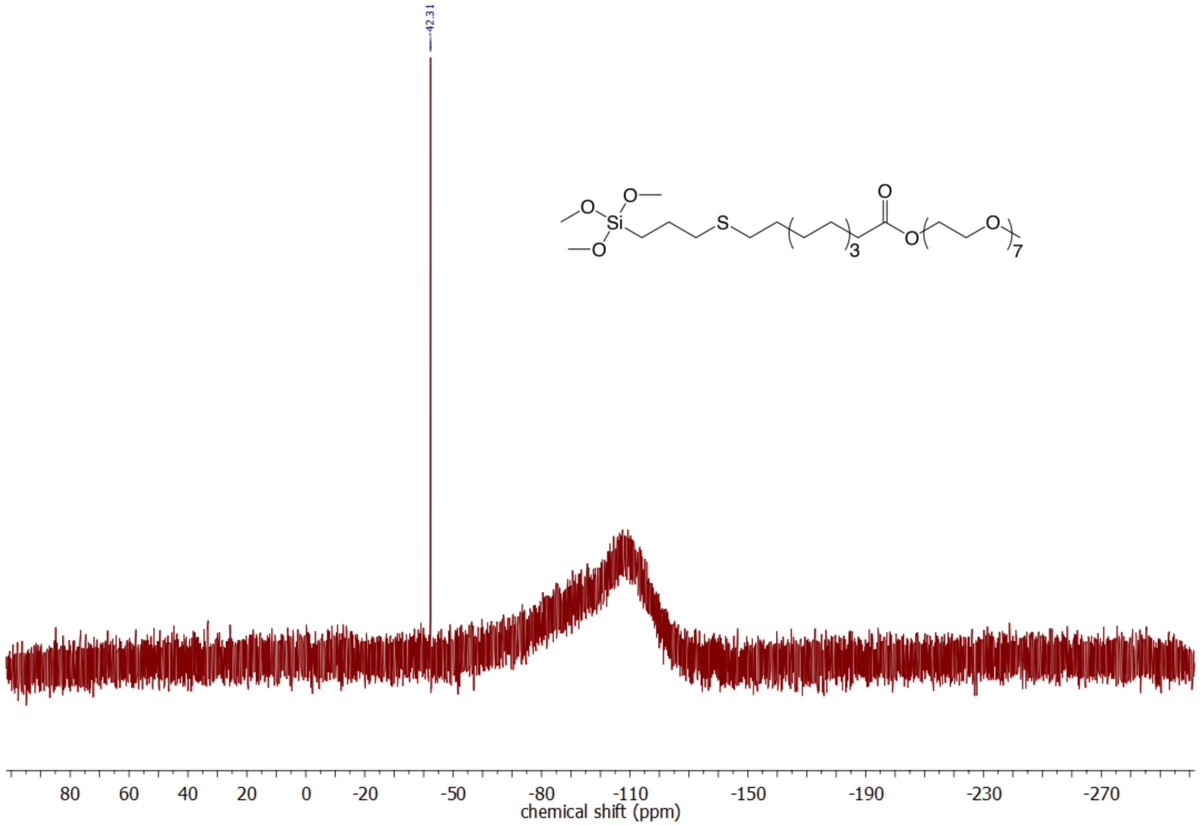

Figure S19. ^{29}Si NMR spectrum of S5

FT-IR spectrum

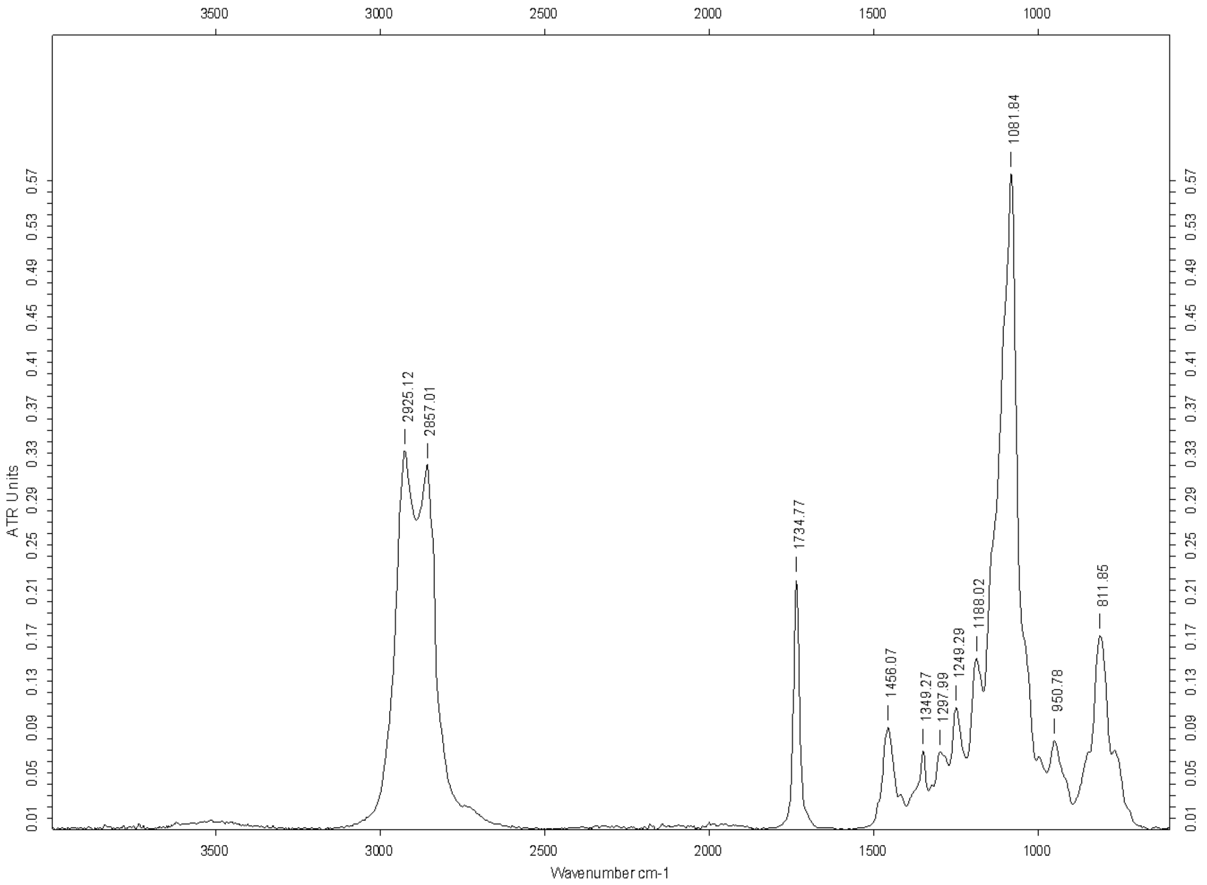

Figure S20. FT-IR spectrum of S5

1.6 S6

Product characterization

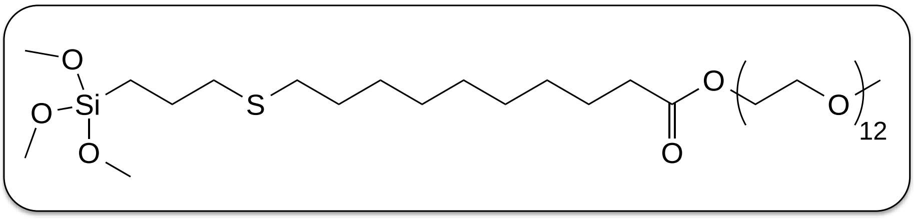

^{1}H NMR (400 MHz, $CDCl_3$): δ 4.16 (m, 2H, $C(O)OCH_2$); 3.59 (m, 48H, CH_2O); 3.51 (s, 9H, $Si(OCH_3)_3$); 3.32 (s, 3H, OCH_3); 2.45 (m, 4H, CH_2SCH_2); 2.26 (t, *J* = 7.6 Hz, 2H, $CH_2C(O)$); 1.62 (dd, *J* = 15.8, 7.6 Hz, 2H, $SiCH_2\underline{CH_2}$); 1.52 (dq, *J* = 22.7, 7.4 Hz, 4H, CH_2); 1.26 (m, 10H, CH_2); 0.70 (t, 2H, $SiCH_2$) ppm. **^{13}C NMR (101 MHz, $CDCl_3$):** δ 173.73 (C=O); 71.91 ($\underline{CH_2}OCH_3$); 70.55 (CH_2O); 69.17 ($C(O)OCH_2\underline{C}H_2$); 63.32 ($C(O)O\underline{C}H_2$); 58.99 ($OCH_3$); 50.49 ($Si(OCH_3)_3$); 35.03 ($SiCH_2CH_2\underline{C}H_2S$); 34.14 $C(O)CH_2$); 31.92, 29.69, 29.30, 29.17, 29.06, 28.86 (CH_2); 24.85 ($C(O)CH_2\underline{C}H_2$); 22.98 ($SiCH_2\underline{C}H_2$); 8.55 ($SiCH_2$) ppm. **^{29}Si NMR (79 MHz, $CDCl_3$):** δ -42,33 ($Si(OCH_3)_3$) ppm.

FT-IR (ATR, cm^{-1}): 3517, 2923, 2859, 1734, 1455, 1349, 1297, 1249, 1188, 1083, 949, 812.

NMR spectra

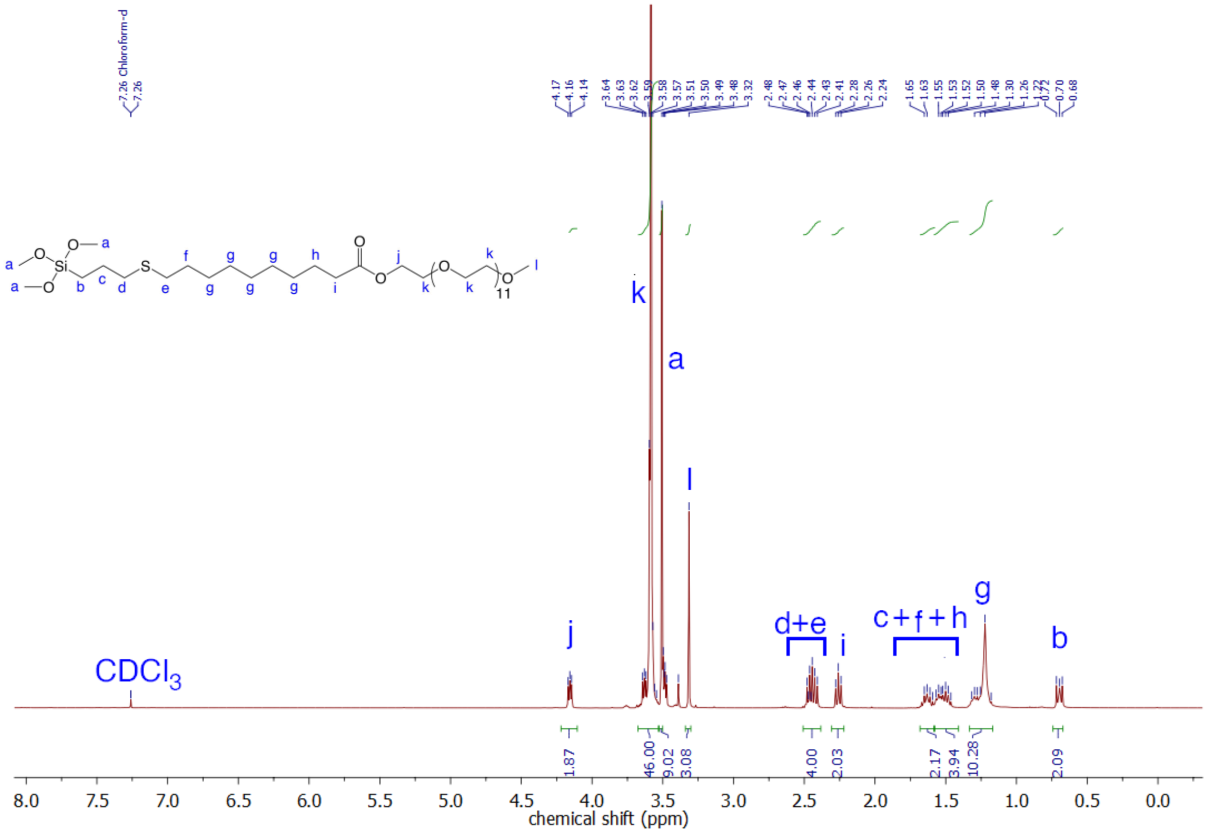

Figure S21. ^{1}H NMR spectrum of S6

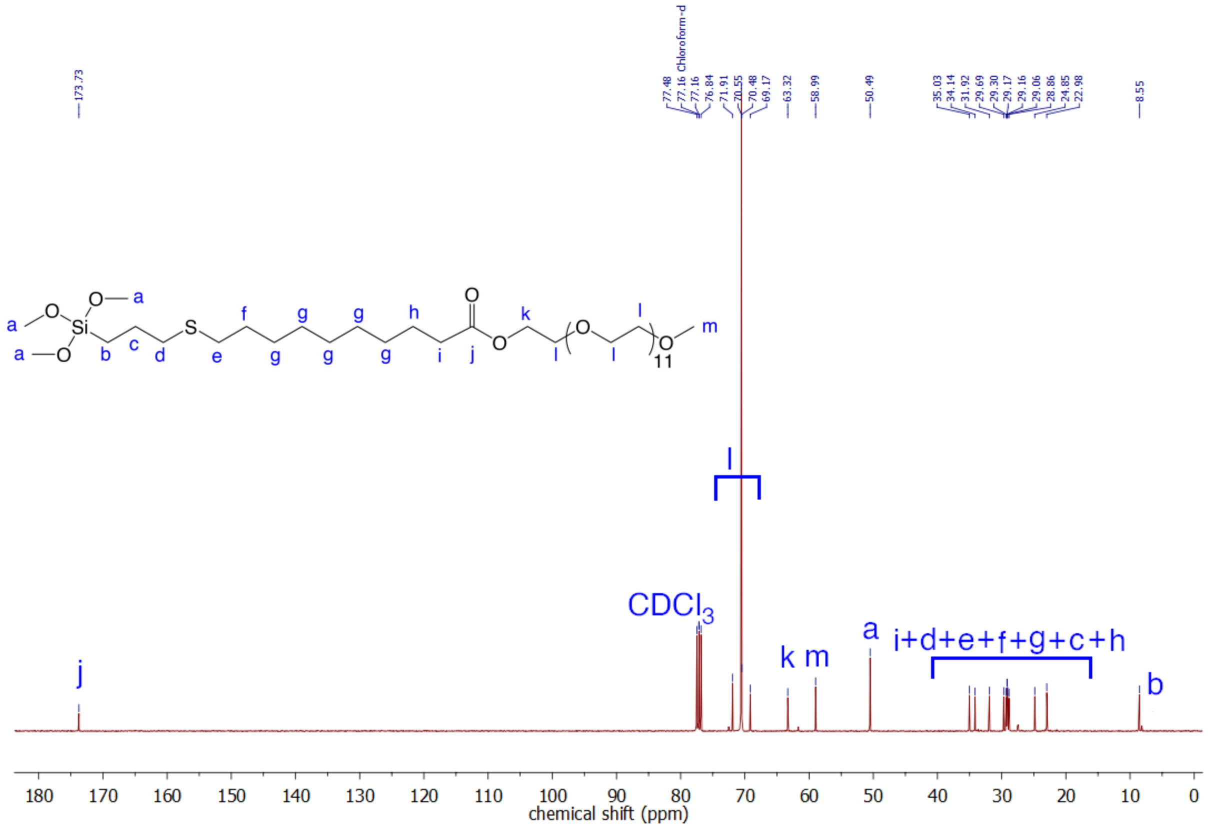

Figure S22. ^{13}C NMR spectrum of S6

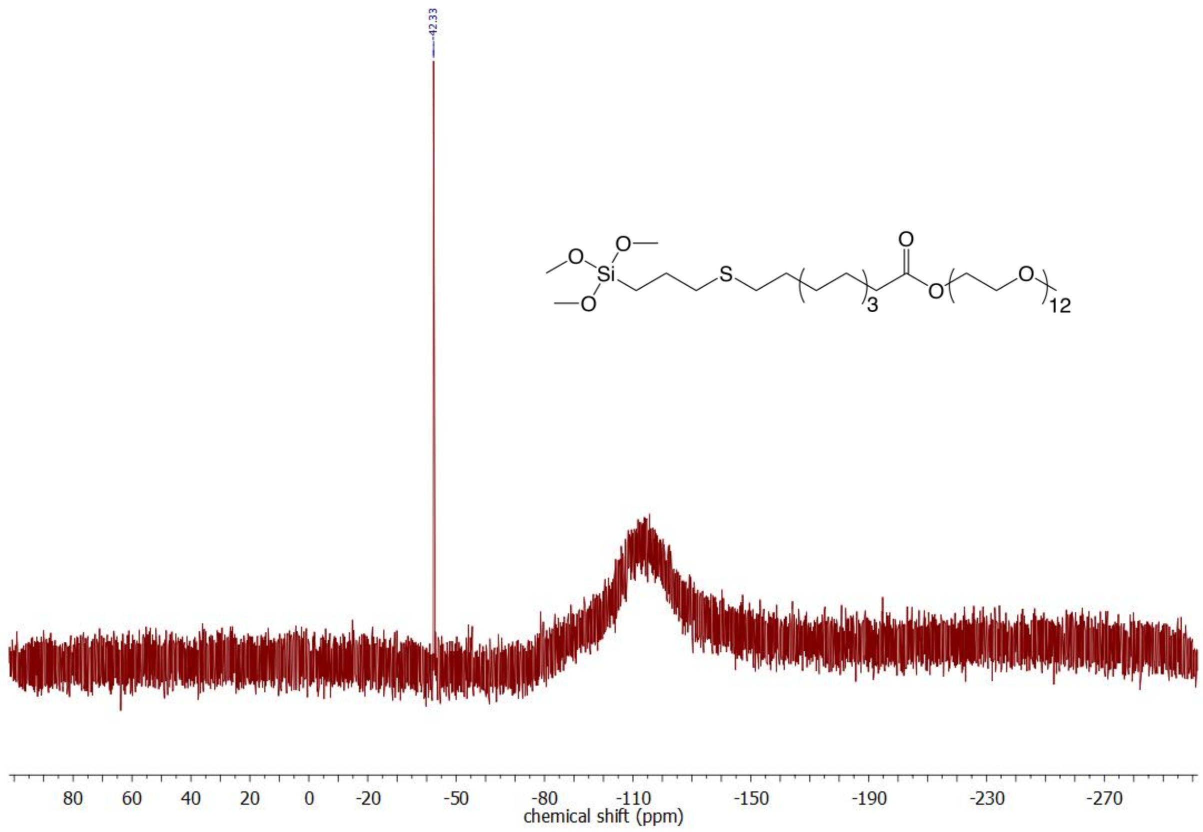

Figure S23. ^{29}Si NMR spectrum of S6

FT-IR spectrum

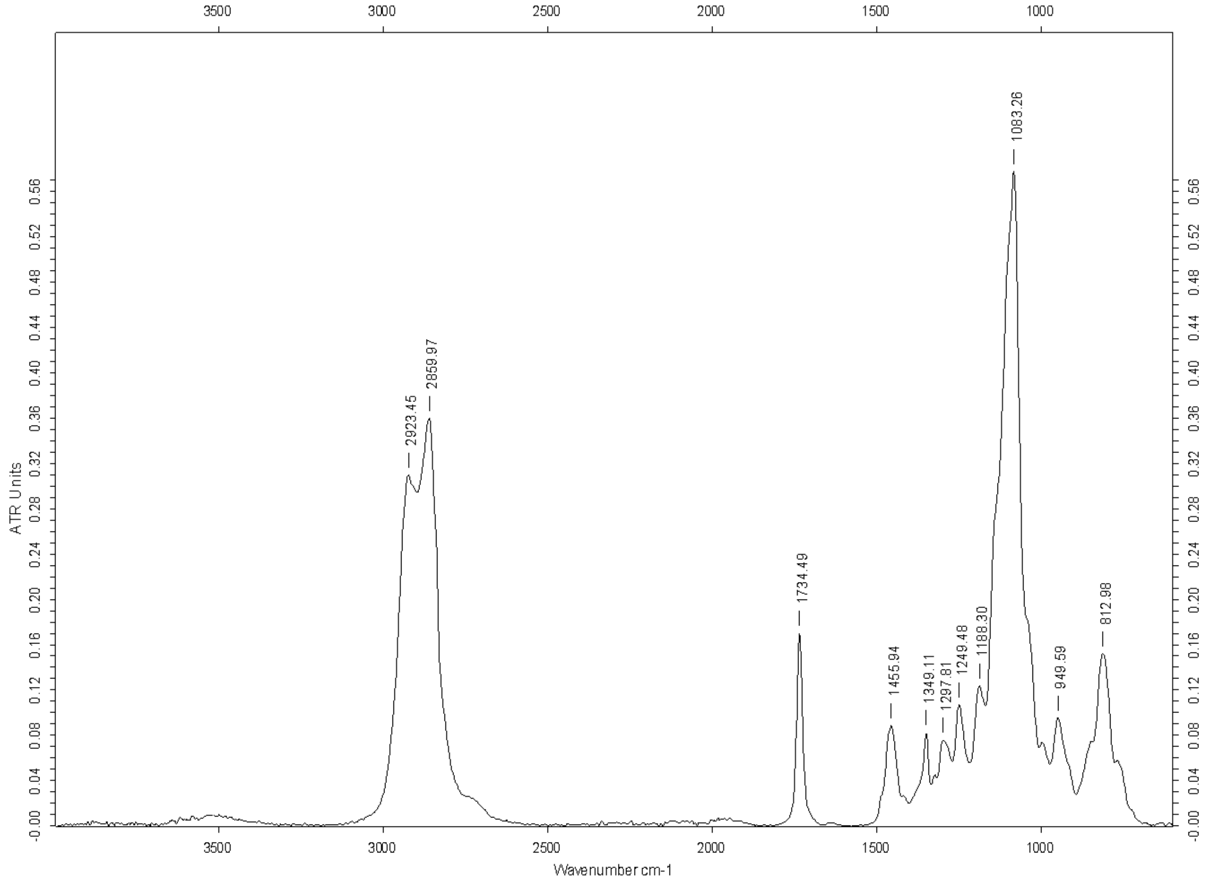

Figure S24. FT-IR spectrum of S6

2. Photographs of glass slides over hot steaming water after the modification (1 day)

	Storage temperature		
Compound	Room temperature	3°C	-20°C
S1	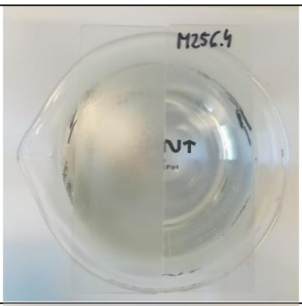	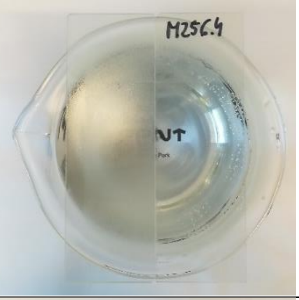	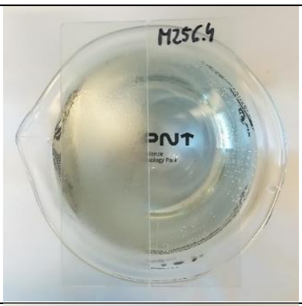
S2	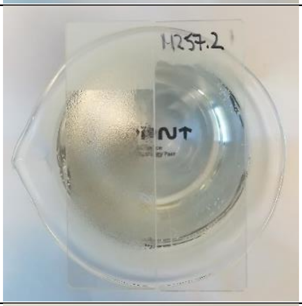	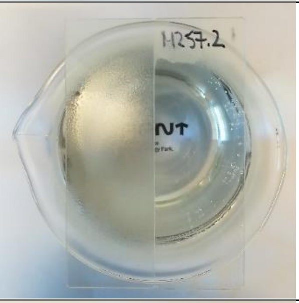	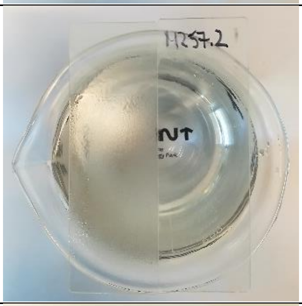
S3	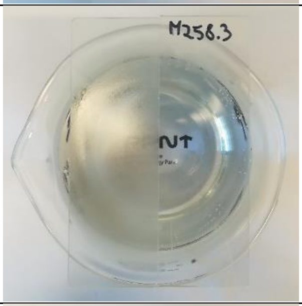	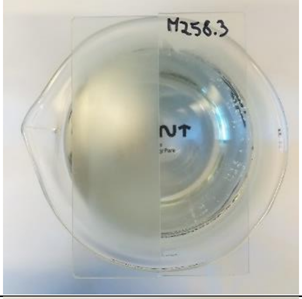	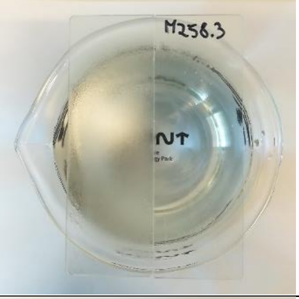
S4	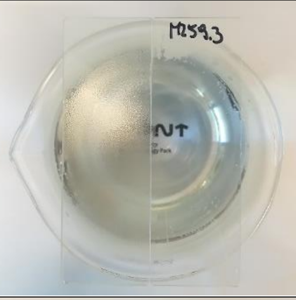	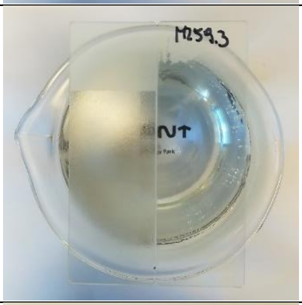	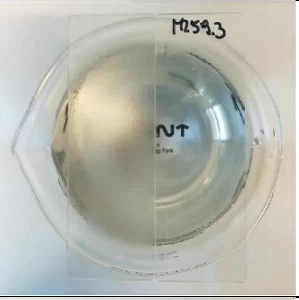
S5	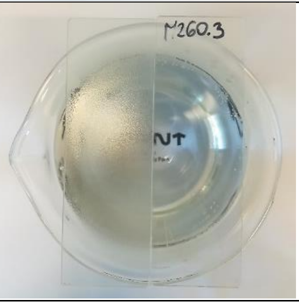	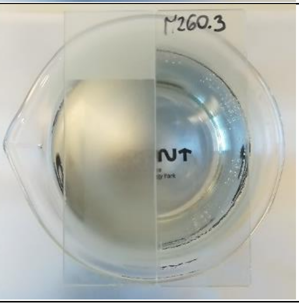	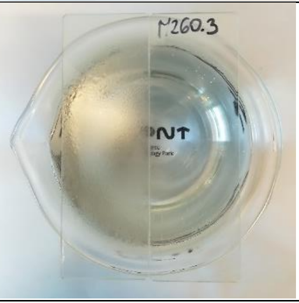

S6	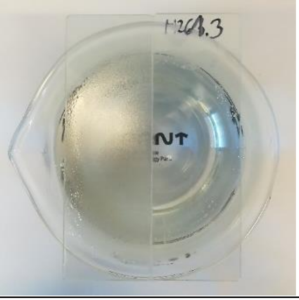	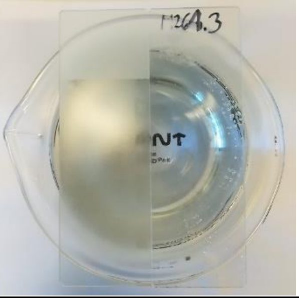	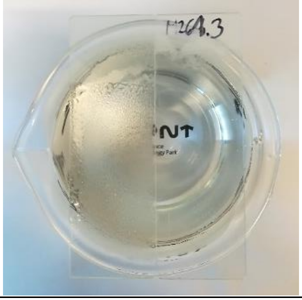

3. Photographs of glass slides over hot steaming water after 3 days of aging the solutions

	Storage temperature		
Compound	Room temperature	3°C	-20°C
S1	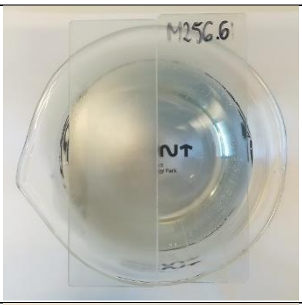	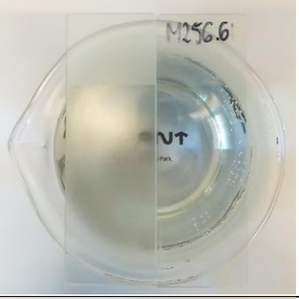	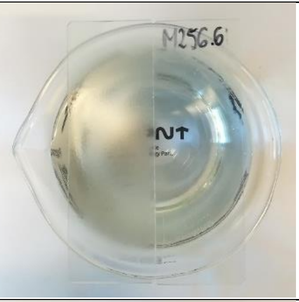
S2	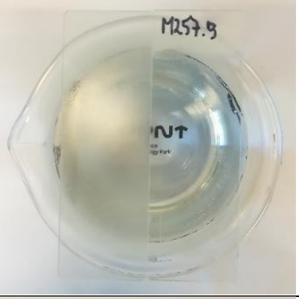	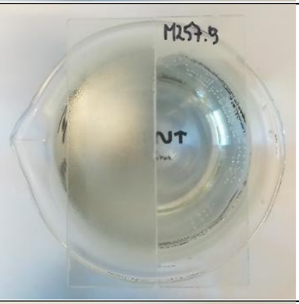	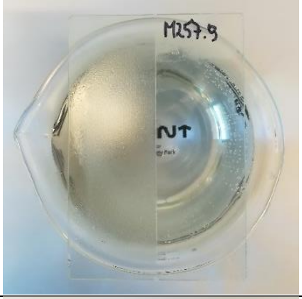
S3	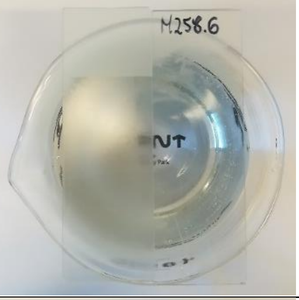	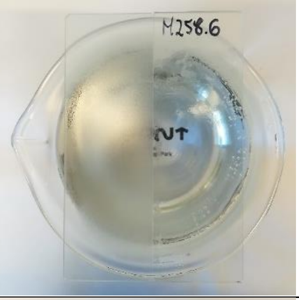	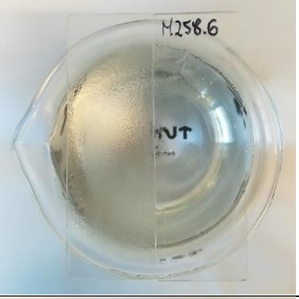
S4	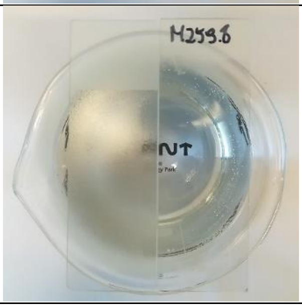	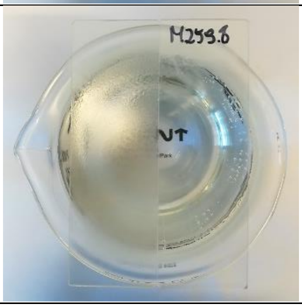	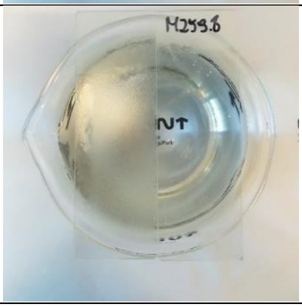

S5	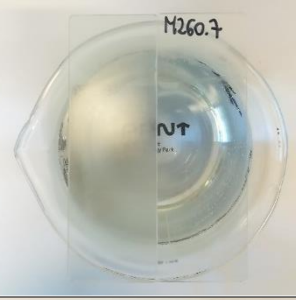	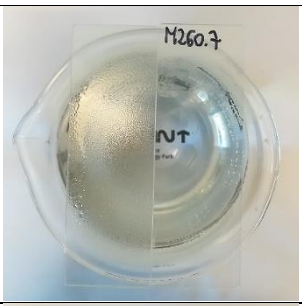	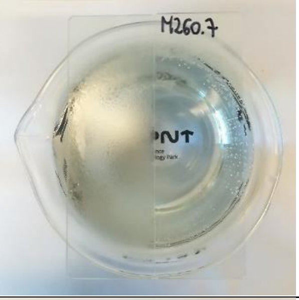
S6	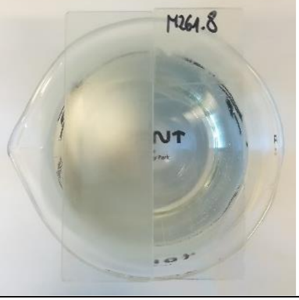	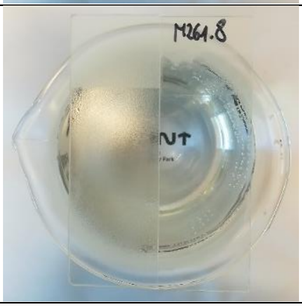	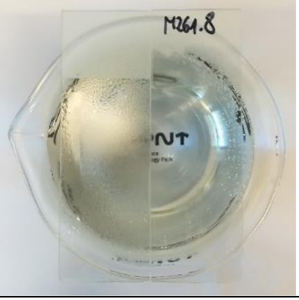

4. Photographs of glass slides over hot steaming water after 5 days of aging the solutions

	Storage temperature		
Compound	Room temperature	3°C	-20°C
S1	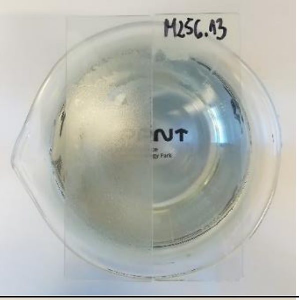	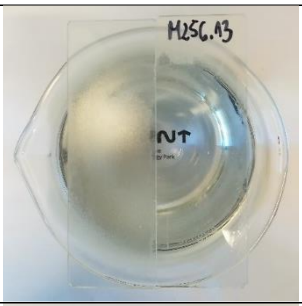	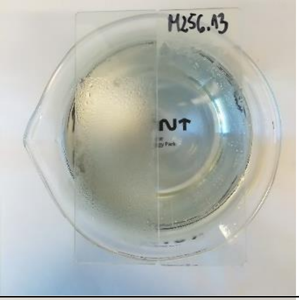
S2	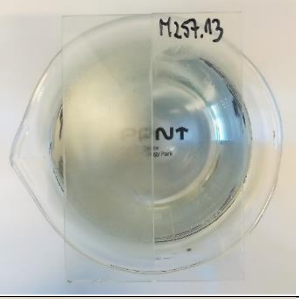	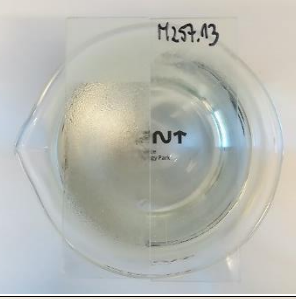	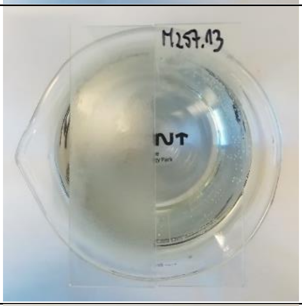
S3	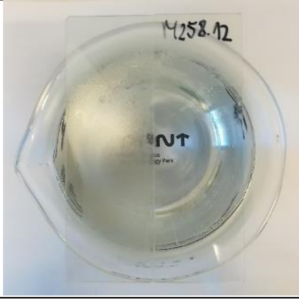	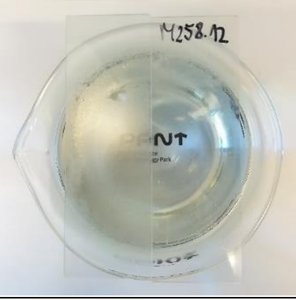	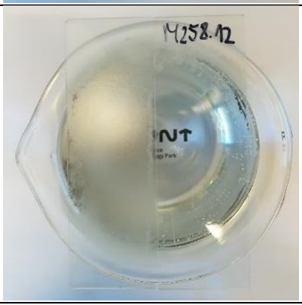

S4	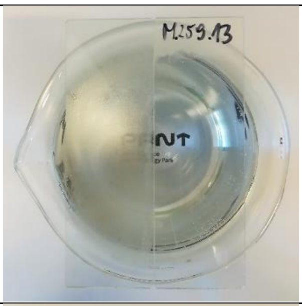	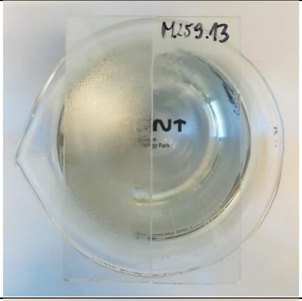	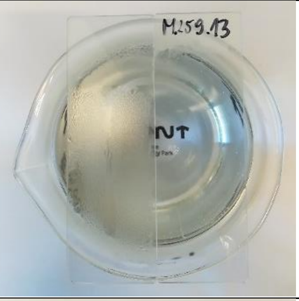
S5	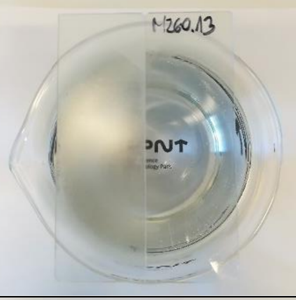	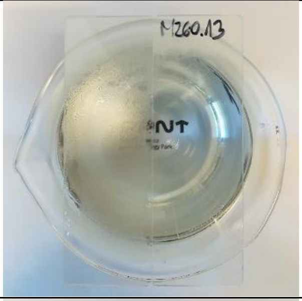	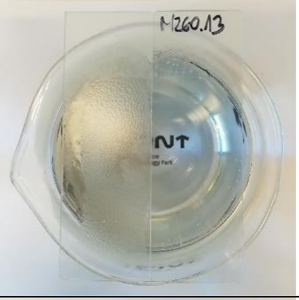
S6	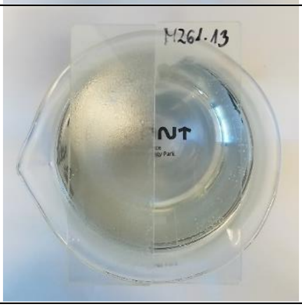	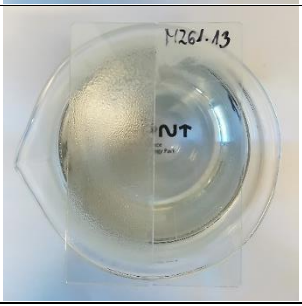	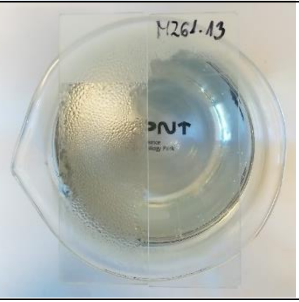

5. Photographs of glass slides over hot steaming water after 10 days of aging the solutions

	Storage temperature		
Compound	Room temperature	3°C	-20°C
S1	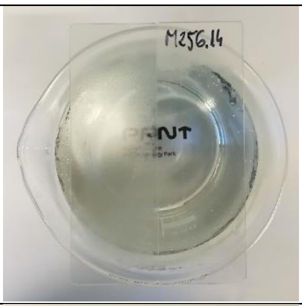	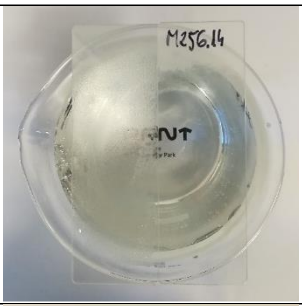	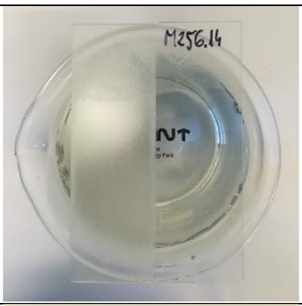
S2	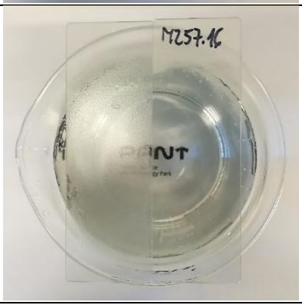	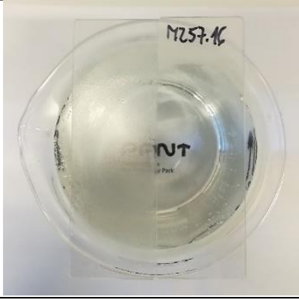	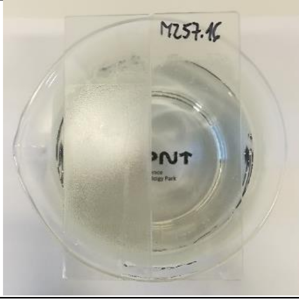

S3	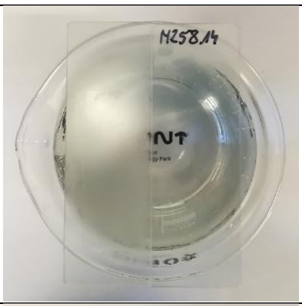	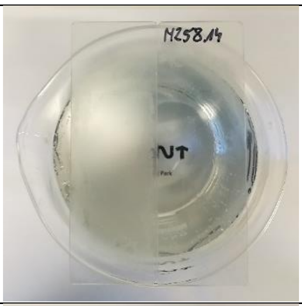	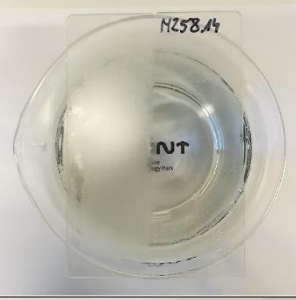
S4	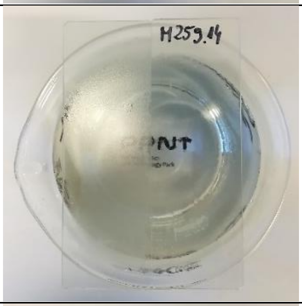	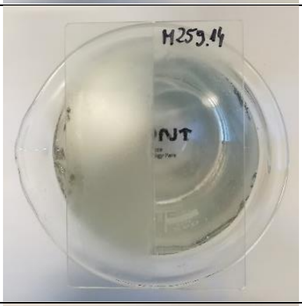	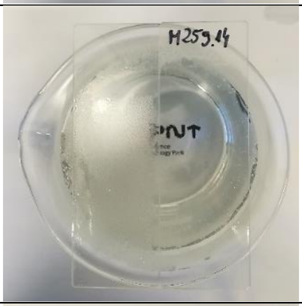
S5	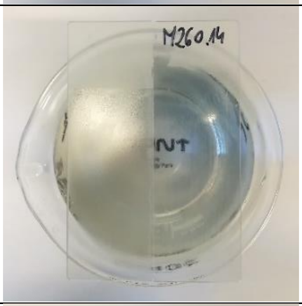	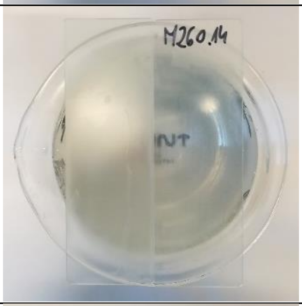	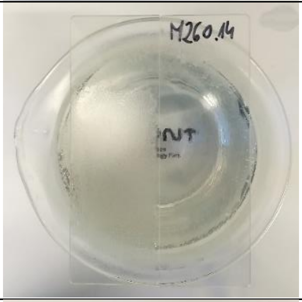
S6	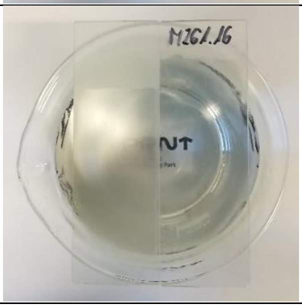	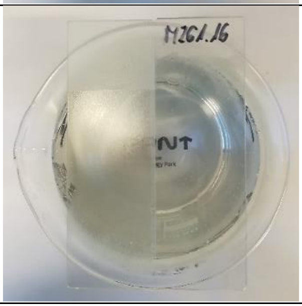	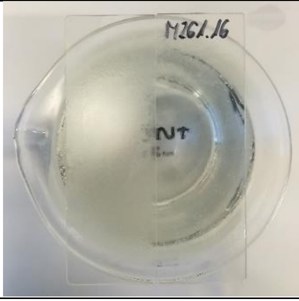

6. Photographs of glass slides over hot steaming water after 20 days of aging the solutions

	Storage temperature		
Compound	Room temperature	3°C	-20°C
S1	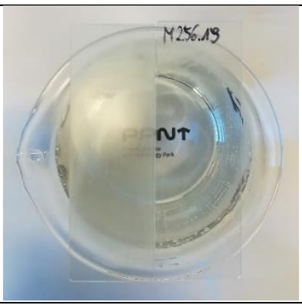	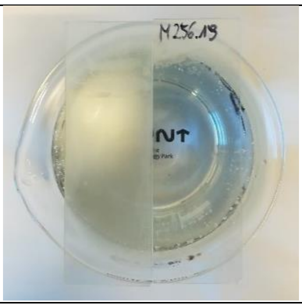	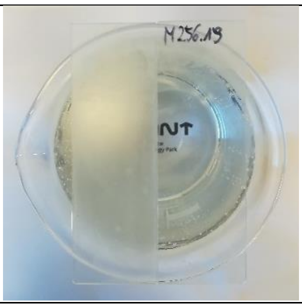

S2	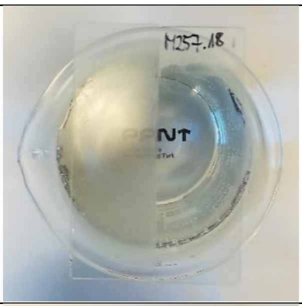M257.18	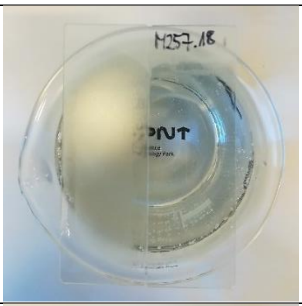M257.18	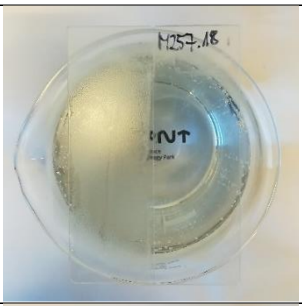M257.18
S3	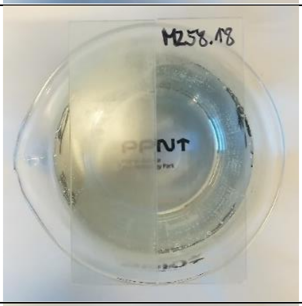M258.18	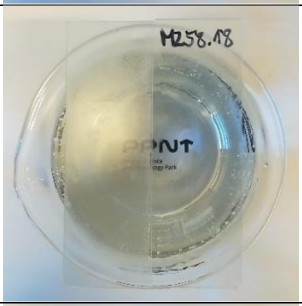M258.18	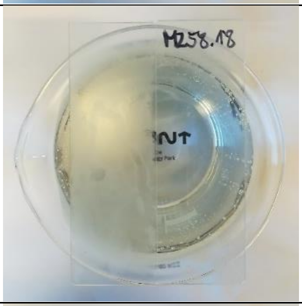M258.18
S4	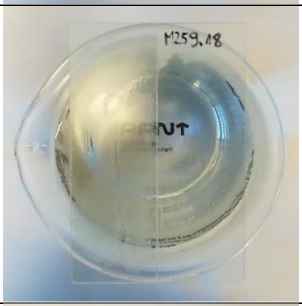M259.18	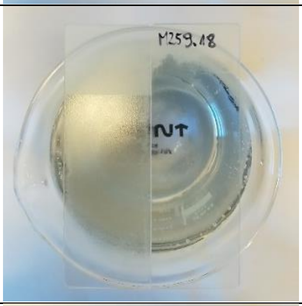M259.18	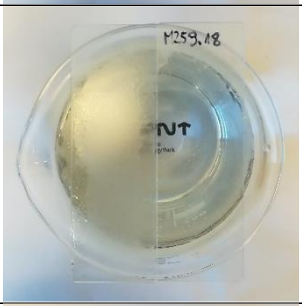M259.18
S5	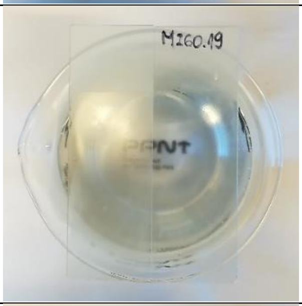M260.19	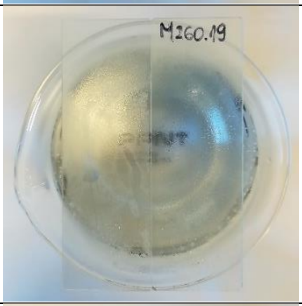M260.19	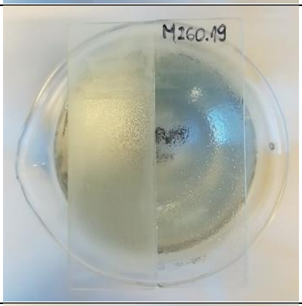M260.19
S6	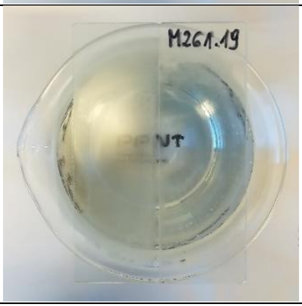M261.19	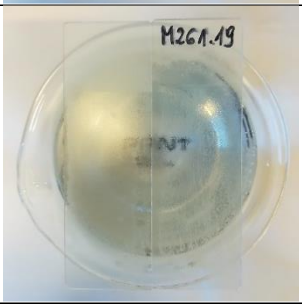M261.19	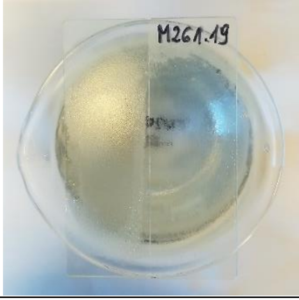M261.19

7. Photographs of glass slides over hot steaming water after 31 days of aging the solutions

	Storage temperature		
Compound	Room temperature	3°C	-20°C
S1	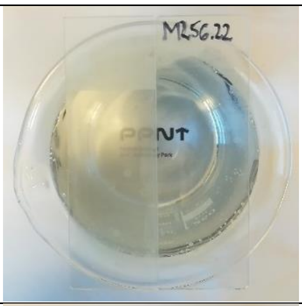	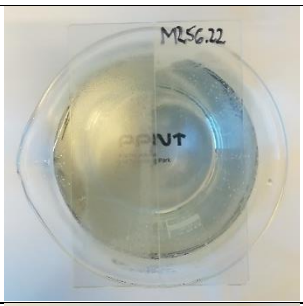	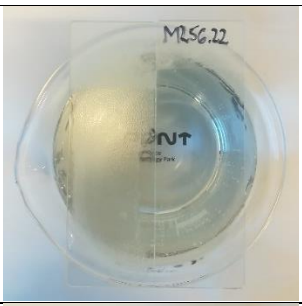
S2	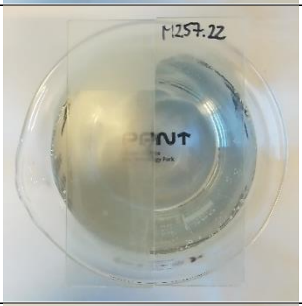	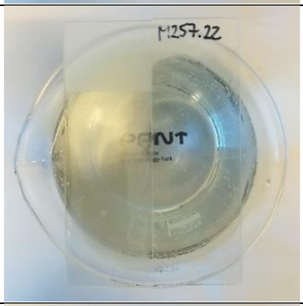	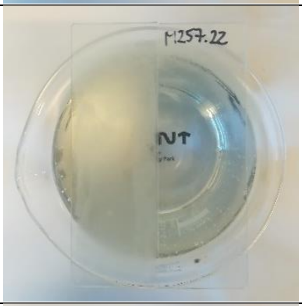
S3	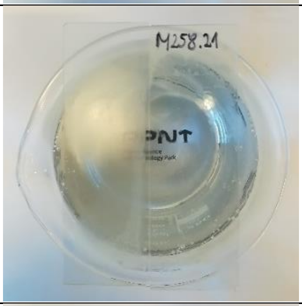	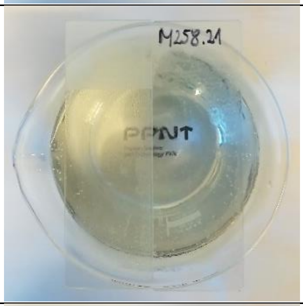	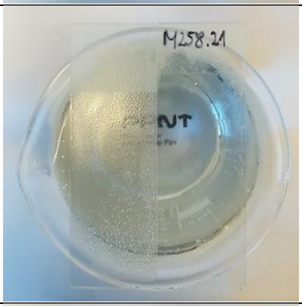
S4	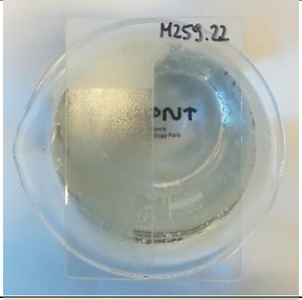	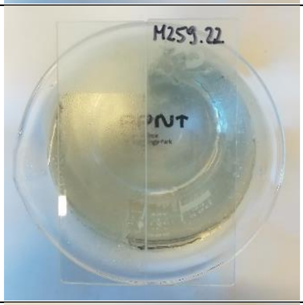	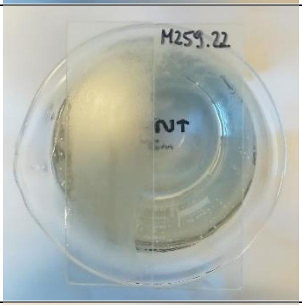
S5	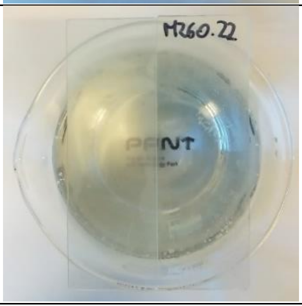	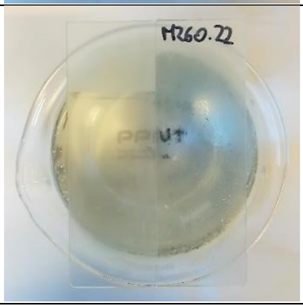	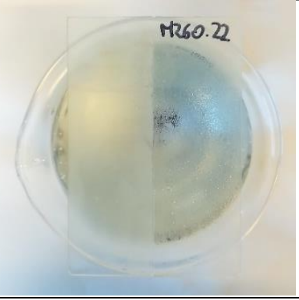

S6	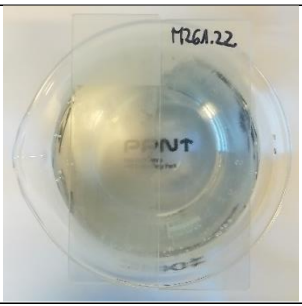	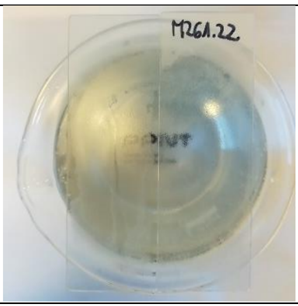	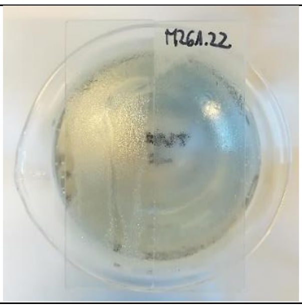

8. Photograph of glass slide over hot steaming water after 8 months of aging S4 solution

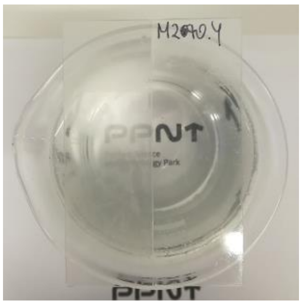

9. WCAs values of coated samples measured after the modification (1 day) and after storage the solutions in a laboratory environment

Compound	WCA [°]					
	1 day	3 days	5 days	10 days	20 days	31 days
S1	8.8±0.3	9.0±0.5	7.8±0.7	8.0±0.2	8.0±0.9	8.2±0.8
S2	8.5±0.2	11.3±0.6	9.1±1.2	9.7±1.0	11.0±0.7	9.9±0.6
S3	8.8±0.6	12.1±0.3	10.4±0.4	9.7±1.5	9.6±0.5	10.1±0.9
S4	11.3±0.6	11.9±0.5	10.5±0.8	7.7±0.3	7.8±0.8	9.8±0.2
S5	42.0±3.9	19.5±1.4	27.6±4.6	0*	0*	0*
S6	38.5±1.6	20.7±1.1	20.7±1.6	8.3±2.4	0*	3.9±0.7

* WCA impossible to measure due to the spreading of the drop on the surface (WCA marked 0°)

10.WCAs values of coated samples measured after storage at 60 °C

Compound	WCA [°]			
	Start	Day 1	Day 14	Day 21
S1	10.1±0.8	8.2±1.4	13.5±2.8	44.3±7.6
S2	9.5±1.0	8.2±1.0	45.3±5.3	45.7±9.2
S3	8.4±1.8	6.7±1.9	6.1±0.5	28.6±19.5
S4	9.5±0.8	9.6±0.8	8.1±1.6	21.3±16.6
S5	23.0±6.8	21.7±6.3	34.9±12.9	29.6±18.5
S6	16.5±5.1	13.2±3.3	29.6±21.7	35.8±17.8

11. WCAs values of coated samples measured after storage at -20 °C

Compound	WCA [°]			
	Start	Day 1	Day 14	Day 21

S1	9.1±1.0	6.1±0.8	8.0±1.7	8.3±1.9
S2	9.3±1.0	6.8±1.7	9.7±2.1	7.6±0.7
S3	8.4±0.3	5.0±1.3	6.7±0.8	4.4±1.4
S4	10.1±1.4	7.3±1.5	7.4±0.7	8.5±3.2
S5	21.6±7.2	19.0±7.0	17.3±4.7	19.6±6.8
S6	15.5±4.0	10.9±4.7	12.7±3.2	10.5±2.5

12. Photographs of glass slides over hot steaming water after 2 months of glass storage

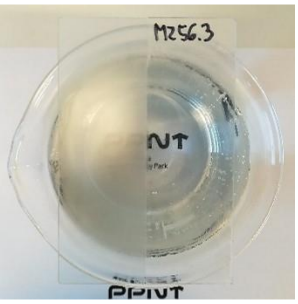	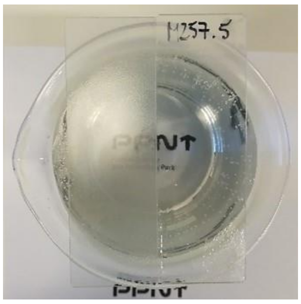	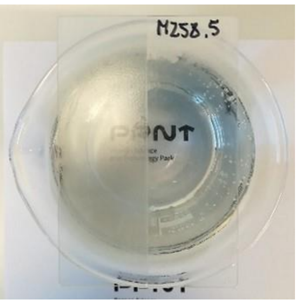
S1	S2	S3
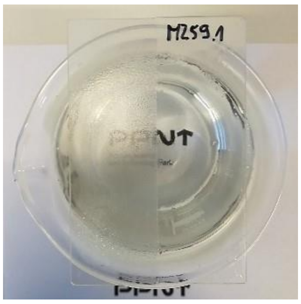	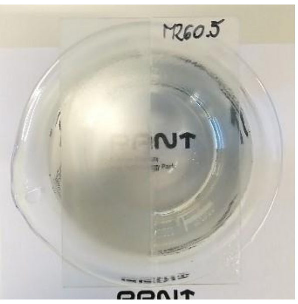	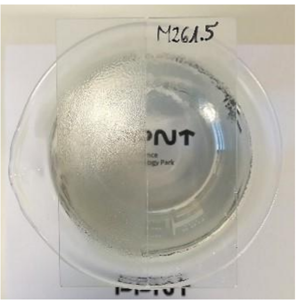
S4	S5	S6

13. Materials of the antifogging coatings

<table>
<tr><th colspan="3">Coating</th><th>WCA</th><th>Light transmission</th><th>Anti-frost</th><th>Anti-fog</th><th>Ref.</th></tr>
<tr><td colspan="3">Zwitterionic coating

silanized zwitterionic sulfobetaine silane (SBSi)</td><td><5°</td><td>>90%</td><td>Yes</td><td>Yes</td><td>[1]</td></tr>
<tr><td rowspan="2">Zwitterionic coatings</td><td rowspan="2">poly(sulfobetaine methacrylate)</td><td>pSBMA-SH</td><td>< 5°</td><td>92%</td><td>Yes</td><td>Yes</td><td rowspan="2">[2]</td></tr>
<tr><td>pSBMA-H</td><td>15 ± 3°</td><td>91%</td><td>No</td><td>No</td></tr>
</table>

	poly(sulfobetaine vinyl imidazole)	pSBVI-SH	< 5°	92%	Yes	Yes	
		pSBVI-H	15 ± 3°	92%	No	No	
Zwitterionic coating reinforced by nanoSiO_2<br><br>*copolymerized sulfobetaine methacrylate and 2-hydroxyethyl methacrylate in the presence of sulfobetaine-modified silica nanoparticles*			Not measured	~90%	Yes	Yes	[3]
Zwitterionic coating<br><br>*(Methacryloyloxy)ethyldimethyl-(3-sulfopropyl) (SBMA) and Itaconic acid (IA)*			8.2°	>95%	Yes	Yes	[4]
POSS-containing coatings<br><br>POSS-P(QAC-*co*-AEMA)<br>*POSS-poly(quaternary ammonium compound-co-2-aminoethyl methacrylate hydrochloride and POSS(HEAA-co-GMA) POSS-poly(N-hydroxyethylacrylamide-co-glycidyl methacrylate blends*			Initial 110° to 54-64° after 400s	85.0-90.0%	Yes	Yes	[5]
POSS-containing coatings<br><br>*POSS-poly[2- (dimethylamino)ethyl methacrylate]-block-poly(sulfobetaine methacrylate) (POSS-PDMAEMA-b-PSBMA) with a ethylene glycol dimethacrylate (EGDMA)*			Initial 105.8° to 43.3° in 60 s and 31.4° in 150 s, (for C-POSS-D_{90}-*b*-S_{13})<br><br>From 60° to about 20° (for C-D_{50}-*b*-S_7)	86-91%	Yes	Yes	[6]
Acrylic coatings<br><br>*Acrylic copolymers of various ratios of 2-(dimethylamino)-ethyl methacrylate and methyl methacrylate, poly(MMA-co-DMAEMA)s dissolved with ethylene glycol dimethacrylate (EGDMA)*			Initial 60-70° decrease by about 13-20° after 600 s	92%	Yes/ No<br><br>Depending on the ratio of monomers and amount of EGDMA in the copolymer	Yes/ No	[7]

Acrylic coatings<br><br>*Acrylic acid (AA), 1H,1H,2H,2H-tridecafluoro-n-octyl acrylate (TFOA), and 4-benzoylphenyl acrylate (BPA)*	Initial ~59° to ~10° within 360 s (for C-5)	~90%	Yes	Yes	[8]
Fluorosilane<br><br>*1H,1H,2H,2H-Perfluorodecyl trichlorosilane*	Initial 110° to 100°	77%	No	No	[7]
TiO_2 thin films	from 19.3° to 1.7°	95.4 to 25.1% (Depending on $NH_3{\cdot}H2O$ concentration)	Not tested	Yes	[9]
TiO_2-PHEA<br><br>*TiO_2/poly(hydroxyethyl acrylate)nanocomposite*	about 3° after 0.45 s	>90%	Not tested	Yes	[10]
SiO_2<br><br>*SiO_2 from TEOS, differed in heating temperature (25–500 °C) (C25-C500)*	From 0° to 56°	91-93.5% (for C400)	Not tested	Yes	[11]
Colloidal SiO_2 with different morphologies	1.7◦ (for optimum coating)	94.8%	Not tested	Yes	[12]
PAA-CMC-($FeCl_3{\cdot}6H_2O$)<br><br>*Mixture of poly(acrylic acid) (PAA), carboxymethyl cellulose (CMC) and iron (III) chloride hexahydrate ($FeCl_3{\cdot}6H_2O$)*	4.6°	90%	Yes	Yes	[13]
PEI-TA<br><br>*Polyethyleneimine/tannic acid complexes*	5°	88.5%	Yes	Yes	[14]
CHINFs/PAA<br><br>*Chitin nanofibers/poly(acrylic acid)*	15°	96.4%	Yes	Yes	[15]
PVA-SA<br><br>*Poly(vinyl alcohol)/salicylic acid*	Initial 60°, ~20-22° after 660 s	>90%	Yes	Yes	[16]

[1] Yu, X. et al. Highly durable antifogging coatings are resistant to long-term airborne pollution and intensive UV irradiation. *Mater. Des.* **194**, 108956 (2020).
[2] Ezzat, M., & Huang, C. J. Zwitterionic polymer brush coatings with excellent anti-fog and anti-frost properties. *RSC Adv.* **6**(66), 61695-61702 (2016).
[3] Liang, B., Zhong, Z., Jia, E., Zhang, G., & Su, Z. Transparent and scratch-resistant antifogging coatings with rapid self-healing capability. *ACS Appl. Mater. Interfaces* **11**(33), 30300-30307 (2019).
[4] Yang, H. et al. Facile preparation of a high-transparency zwitterionic anti-fogging poly (SBMA-co-IA) coating with self-healing property. *Prog. Org. Coat.* **165**, 106764 (2022).
[5] Bai, S., Li, X., Zhao, Y., Ren, L., & Yuan, X. Antifogging/antibacterial coatings constructed by N-hydroxyethylacrylamide and quaternary ammonium-containing copolymers. *ACS Appl. Mater. Interfaces* **12**(10), 12305-12316 (2020).
[6] Li, C. et al. Amphiphilic antifogging/anti-icing coatings containing POSS-PDMAEMA-b-PSBMA. *ACS Appl. Mater. Interfaces* **9**(27), 22959-22969 (2017).
[7] Zhao, J., Meyer, A., Ma, L., & Ming, W. Acrylic coatings with surprising antifogging and frost-resisting properties. *ChemComm* **49**(100), 11764-11766 (2013).
[8] Xu, J. et al. UV curable stimuli-responsive coatings with antifogging and oil-repellent performances. *J. Mater. Chem. A* **9**(46), 26028-26035 (2021).
[9] Li, H., Li, N., Zhang, Y., He, H., & Liu, Z. Visible light-induced super-hydrophilic anatase porous thin film with easy-to-clean and antifogging properties *JSST* **83**, 502-517 (2017).
[10] Zhang, Y., Zhang, S., & Wu, S. Room-temperature fabrication of TiO2-PHEA nanocomposite coating with high transmittance and durable superhydrophilicity. *Chem. Eng. J.* **371**, 609-617 (2019).
[11] Ye, L., Zhang, Y., Song, C., Li, Y., & Jiang, B. A simple sol-gel method to prepare superhydrophilic silica coatings. *Mater. Lett.* **188**, 316-318 (2017).
[12] Li, N., Kuang, J., Ren, Y., Li, X., & Li, C. Fabrication of transparent super-hydrophilic coatings with self-cleaning and anti-fogging properties by using dendritic nano-silica. *Ceram. Int.* **47**(13), 18743-18750 (2021).
[13] Wang, X. et al. A multifunctional and environmentally-friendly method to fabricate superhydrophilic and self-healing coatings for sustainable antifogging. *Chem. Eng. J.* **409**, 128228 (2021).
[14] Ren, J., Kong, R., Gao, Y., Zhang, L., & Zhu, J. Bioinspired adhesive coatings from polyethylenimine and tannic acid complexes exhibiting antifogging, self-cleaning, and antibacterial capabilities. *J. Colloid Interface Sci.* **602**, 406-414 (2021).
[15] Manabe, K. et al. Chitin nanofibers extracted from crab shells in broadband visible antireflection coatings with controlling layer-by-layer deposition and the application for durable antifog surfaces. *ACS Appl. Mater. Interfaces* **8**(46), 31951-31958 (2016).
[16] Wang, W. et al. A facile antifogging/frost-resistant coating with self-healing ability. *Chem. Eng. J.* **378**, 122173 (2019).
